# Diverse nucleotide substitutions in rice base editing mediated by novel TadA variants

**DOI:** 10.1016/j.xplc.2024.100926

**Published:** 2024-05-08

**Authors:** Man Yu, Yongjie Kuang, Chenyang Wang, Xuemei Wu, Shaofang Li, Dawei Zhang, Wenxian Sun, Xueping Zhou, Bin Ren, Huanbin Zhou

**Affiliations:** 1 State Key Laboratory for Biology of Plant Diseases and Insect Pests, Institute of Plant Protection, Chinese Academy of Agricultural Sciences, Beijing 100193, China; 2 Department of Plant Pathology, China Agricultural University, Beijing 100193, China; 3 Scientific Observing and Experimental Station of Crop Pests in Guilin, Ministry of Agriculture and Rural Affairs, Guilin 541399, China; 4 Ministry of Education Key Laboratory for Bio-Resource and Eco-Environment, College of Life Science, State Key Laboratory of Hydraulics and Mountain River Engineering, Sichuan University, Chengdu 610064, China; 5 State Key Laboratory of Vegetable Biobreeding, National Engineering Research Center for Vegetables, Beijing Vegetable Research Center, Beijing Academy of Agriculture and Forestry Science, Beijing 100097, China; 6 State Key Laboratory of Rice Biology, Institute of Biotechnology, Zhejiang University, Hangzhou 310058, China; 7 Key Laboratory of Gene Editing Technologies (Hainan), Ministry of Agricultural and Rural Affairs, Sanya 572024, China

**Keywords:** CRISPR, TadA variants, cytosine base editing, dual base editor, rice

## Abstract

CRISPR-mediated base editors have been widely used to correct defective alleles and create novel alleles by artificial evolution for the rapid genetic improvement of crops. The editing capabilities of base editors strictly rely on the performance of various nucleotide modification enzymes. Compared with the well-developed adenine base editors (ABEs), cytosine base editors (CBEs) and dual base editors suffer from unstable editing efficiency and patterns at different genomic loci in rice, significantly limiting their application. Here, we comprehensively examined the base editing activities of multiple evolved TadA8e variants in rice. We found that both TadA-CDd and TadA-E27R/N46L achieved more robust C-to-T editing than previously reported hyperactive hAID∗Δ, and TadA-CDd outperformed TadA-E27R/N46L. A C-to-G base editor (CGBE) engineered with TadA-CDd and OsUNG performed highly efficient C-to-G editing in rice compared with that of TadA-N46P. In addition, a dual base editor constructed with a single protein, TadDE, enabled simultaneous, highly efficient C-to-T and A-to-G editing in rice. Collectively, our results demonstrate that TadA8e derivatives improve both CBEs and dual base editors in rice, providing a powerful way to induce diverse nucleotide substitutions for plant genome editing.

## Teaser

This study introduces the using of TadA variants to create a series of novel rice base editors that can achieve highly efficient C-to-T, C-to-G, and simultaneous A-to-G and C-to-T editing in the rice genome, providing a foundation for gene functional research and molecular breeding in plants.

## Introduction

Rice is a crucial global food crop that serves as a major food source for the world’s population. SNPs are a common form of genetic diversity in crops and are closely associated with numerous agronomic traits. The exploration and utilization of specific SNPs thus facilitates genetic improvement and expedites the breeding process. CRISPR-mediated base editors can introduce single-nucleotide substitutions at target genomic sites without the DNA double-strand break and repair template and have been widely used to correct defective alleles and create novel alleles, thus greatly accelerating functional annotation, directed evolution, and genetic improvement of target genes in crops (Ren et al., 2018; Kuang et al., 2020; Li et al., 2020; Gao, 2021; Molla et al., 2021; Yan et al., 2021; Wang et al., 2022a; Zhang et al., 2023a, 2023b). Currently, several base editors, including cytosine base editors (CBEs), adenine base editors (ABEs), dual base editors, C-to-G base editors (CGBEs), and A-to-K base editors (AKBEs), have been successfully developed in plants and typically consist of a deficient Cas nuclease and single-stranded DNA-specific deaminases or glycosylases (Zong et al., 2017; Yan et al., 2018; Li et al., 2020; Tian et al., 2022; Wu et al., 2023).

Among the many types of base editors, CBEs and ABEs are the two major types that have been most extensively studied, and they are already widely used for crop genetic improvement. They are both chimeric proteins in which a Cas9 nickase is fused with an engineered cytosine or adenine deaminase. They catalyze the deamination of cytosine (C) and adenine (A) as a crucial step to produce uridine (U) and inosine (I) intermediates, which are in turn transformed into thymine (T) and guanine (G) by endogenous DNA repair or replication mechanisms (Komor et al., 2016; Nishida et al., 2016; Gaudelli et al., 2017; Zong et al., 2017; Ren et al., 2018; Yan et al., 2018). Uracil glycosylase inhibitor (UGI) is also fused to the C terminus of CBEs to inhibit glycosylation and base excision of U and ultimately increase C-to-T editing (Komor et al., 2016; Ren et al., 2018). More recently, new CGBEs have been developed from CBEs in rice by replacing UGI with uracil DNA N-glycosylase (UNG) to enable efficient C-to-G base transversion (Tian et al., 2022; Zeng et al., 2022). Greatly improved ABEs that enable highly efficient A-to-G conversions have been developed using highly active and laboratory-engineered adenine deaminases such as TadA8e and TadA9, together with SpCas9 nuclease (Yan et al., 2021). By contrast, although CBEs have been constantly upgraded using various natural cytosine deaminases and engineered variants with different deamination activities (e.g., PmCDA1, hAID, hAPOBEC3A, Anc689, and FERNY) (Ren et al., 2018; Zong et al., 2018; Zeng et al., 2020; Tian et al., 2022; Zhang et al., 2023a, 2023b), they still exhibit significantly variable editing efficiency (and even an inability to edit) at different genomic sites in plants. Therefore, it remains necessary to develop novel plant CBEs and CGBEs with high editing activity for any arbitrary target sequence in the plant genome.

Many agriculturally important traits are associated with multiple heterogeneous nucleotide substitutions. Development of an effective dual base editor is highly desirable for the directed evolution of target genes with multiple heterogeneous nucleotide substitutions in crops. Dual base editors, in which both cytosine and adenine deaminases are fused to a single Cas protein, have been developed for performing simultaneous C-to-T and A-to-G conversions in plants (Li et al., 2020; Xu et al., 2021a; Wang et al., 2022a; Zhang et al., 2023a, 2023b). However, the mutagenesis capacity and editing windows of cytosine and adenine deaminases are more or less impaired in dual-function base editors compared with single-function base editors owing to preferences for C- or N-terminal fusion, conformational changes, and competition within the editable window (Li et al., 2020; Wang et al., 2022a; Zhang et al., 2023a, 2023b). Therefore, new deaminases or strategies are needed to enable multiple heterogeneous nucleotide substitutions.

Recent studies have shown that the highly active adenine deaminase TadA8e can be artificially transformed into a cytosine deaminase and that the engineered TadA variants confer highly efficient C-to-T editing in human cells (Chen et al., 2023; Neugebauer et al., 2023). However, whether these variants are suitable for optimization of plant CBEs and relevant base editors has not yet been investigated. Here, we comprehensively evaluated the efficacy of several evolved TadA variants, TadA-CDd (Neugebauer et al., 2023), TadA-E27R/N46L (Chen et al., 2023), and TadA-N46P (Chen et al., 2023), for cytosine editing in rice and found that TadA-CDd-mediated CBEs and CGBEs performed highly efficient C-to-T and C-to-G editing, respectively. In addition, we developed a new version of the dual base editor using TadDE (Neugebauer et al., 2023) that could perform simultaneous C-to-T and A-to-G editing in rice. These findings demonstrate that TadA-derived rice base editors provide powerful operational tools for precise crop breeding and a potential screening platform for direct evolution within plants.

## Results and discussion

### Novel CBEs derived from TadA variants improve cytosine base editing efficiency in rice

**Figure 1:**
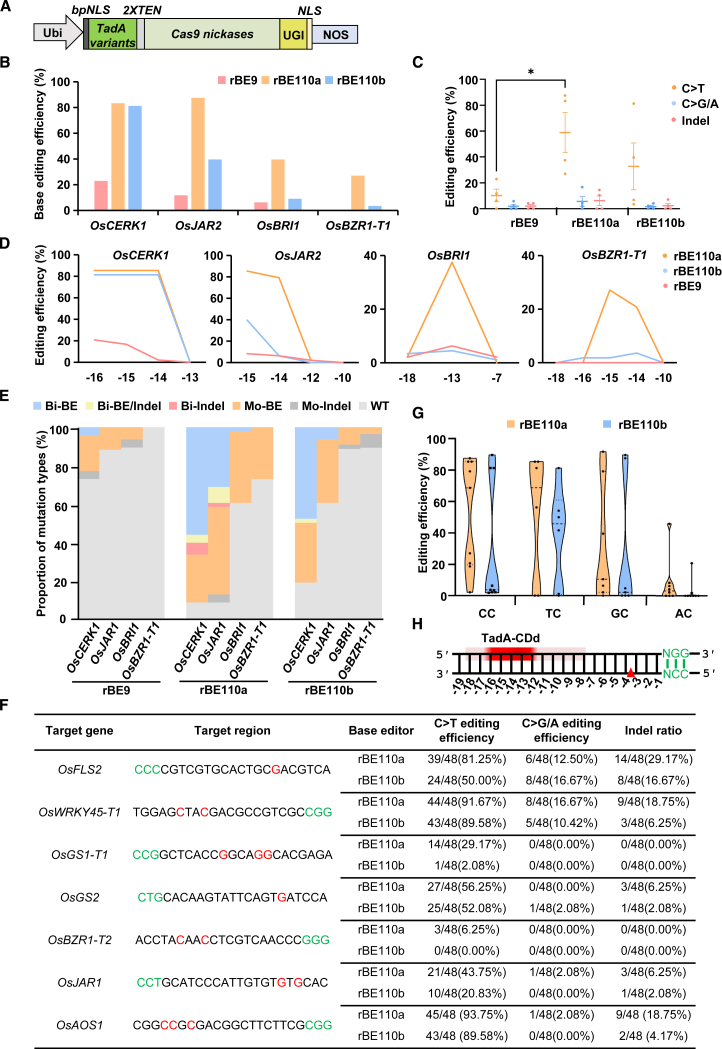
Characterization of TadA-variant-mediated CBEs with SpCas9n in rice. **(A)** Schematic illustration of TadA-variant-mediated cytosine base editors (CBEs) with SpCas9n in rice. Ubi-P, maize ubiquitin 1 promoter; TadA variants, engineered TadA-CDd and TadA-E27R/N46L genes; UGI, uracil DNA glycosylase inhibitor; NLS, nuclear localization sequence; NOS, nopaline synthase terminator. **(B)** Comparison of cytosine editing efficiencies of rBE9, rBE110a, and rBE110b at four target sites in T_0_ transgenic rice lines. **(C)** Summary of editing activities induced by rBE9, rBE110a, and rBE110b. **(D)** Frequencies of cytosine editing within the target regions of four target sites in T_0_ transgenic rice lines using various CBE tools. **(E)** Proportion of mutation types induced by rBE9, rBE110a, and rBE110b at four target sites. Bi, bi-allelic mutations; Mo, mono-allelic mutations; WT, wild type. **(F)** Summary of the base editing efficiencies of rBE110a and rBE110b at seven tested genomic sites in transgenic rice lines. The PAM sequences and targeted nucleotides are highlighted in green and red, respectively. **(G)** Base editing efficiencies of the rBE110a and rBE110b systems at the target C in different sequence contexts. **(H)** The activity windows of TadA-CDd-mediated cytosine base editing. The activity window, predicted to be between positions −16 and −12, is highlighted in red (darker red indicates a higher editing efficiency; lighter red denotes a lower editing efficiency); the PAM sequence is shown in green, and the nick site in the target DNA backbone for SpCas9 nickase is denoted by a red triangle; numbers indicate the positions within the target region.

To explore whether potential TadA variants can improve the efficiency of cytosine base editing in plants, we first evaluated their cytosine deaminase activity in rice. Two engineered TadA variants, *TadA-CDd* carrying the E27K/V28A/M61I/H96N mutation set and *TadA-E27R/N46L*, were selected for their relatively high cytosine base editing activity and wider editing window in human cells (Chen et al., 2023; Neugebauer et al., 2023). They were connected at both ends of *SpCas9n-UGI* with an XTEN linker and a nuclear localization signal (NLS) sequence after codon optimization for expression in rice, resulting in the chimeric genes *TadA-CDd-SpCas9n-UGI* (*rBE110a*) and *TadA-E27R/N46L-SpCas9n-UGI* (*rBE110b*), respectively (Figure 1A; Supplemental Table 1). We selected four targets that had shown low editing efficiency with the previously reported representative rBE9 (*hAID∗Δ-SpCas9n-UGI*) system, individually targeting *OsCERK1*, *OsJAR2*, and *OsBRI1* via NGG PAM and *OsBZR1-T1* via NAG PAM (Ren et al., 2018; unpublished data), to test the performance of rBE110a and rBE110b in transgenic rice using the same single guide RNA (sgRNA) targeting sites as in earlier work. Genotyping of independent T_0_ transgenic rice lines indicated that rBE110a and rBE110b generated the expected C-to-T conversions at all four target sites, and the efficiencies of rBE110a and rBE110b were significantly higher than that of rBE9 (Figure 1B). In the case of the rBE9-resistant target *OsBZR1-T1* site, rBE110b yielded few but detectable C-to-T conversions, whereas rBE110a resulted in substantially improved C-to-T editing (Figure 1B). In addition to the C-to-T conversions, a small number of C-to-G/A and indel (insertion and deletion) editing events were also induced by rBE110a and rBE110b (Figure 1C; Supplemental Figures 1–4). The majority of indel mutations were C-to-indel mutations that occurred in the editing window and resulted from unfaithful base excision repair (BER) of the uracil (Supplemental Figures 1–4). For the four tested target sites, rBE110a exhibited a modestly expanded editing window (−16 to −13) compared with those (−16 to −14) of rBE110b and rBE9 (Figure 1D; Supplemental Figures 1–4). Moreover, we found that rBE110a and rBE110b generated more bi-allelic mutations with only base editing than rBE9 (Figure 1E). These data indicate that rBE110a and rBE110b enable highly efficient C-to-T editing in the rice genome and that the evolved TadA-CDd outperforms TadA-E27R/N46L for increasing CBE activity in rice.

To examine the sequence context preferences of rBE110a and rBE110b, we tested seven additional genomic sites in transgenic rice. The results showed that the base editing efficiency of rBE110a was still higher than that of rBE110b, with the same trend of C-to-G/A and indels (Figure 1F; Supplemental Figures 5–11). Whereas no obvious sequence context preference was observed in TadA-mediated ABEs in rice, the TadA-variant-mediated CBEs exhibited a degree of sequence context preferences, and cytosine base editing activity followed the order CC > TC > GC > AC (Figure 1G). Thus the diminished efficiency of rBE110a and rBE110b at the *OsBZR1-T2* target site might reflect the sequence context preferences of TadA-variant-mediated CBE, as well as other influential factors such as DNA secondary structures and modification status near the intended target site. When examining all the data, we deduced that the activity window of TadA-CDd-based rBE110a was approximately 5 bp, spanning from −16 to −12 bp upstream of the PAM (Figure 1H; Supplemental Figures 1–12), slightly narrower than the 7 bp (−18 to −12 bp) observed in mammals (Neugebauer et al., 2023). The difference in editing window length reflects the complexities of cytosine deamination and DNA replication and repair in different organisms. Combined, these data indicate that the artificially engineered cytosine deaminase TadA-CDd can improve cytosine base editing efficiency in rice.

### TadA variants are compatible with SpRY for efficient cytosine base editing

**Figure 2:**
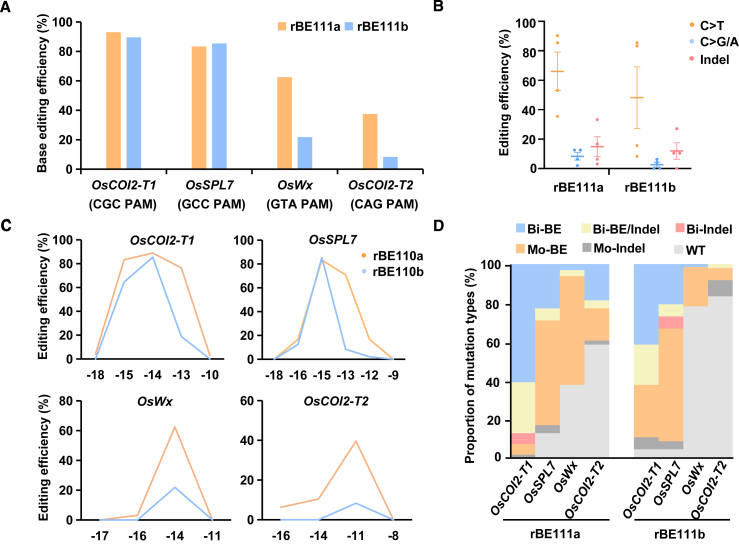
SpRYn-guided TadA variants enable efficient C-to-T editing by recognizing a highly flexible PAM in rice. **(A)** Comparison of cytosine editing efficiencies of SpRYn-guided rBE111a and rBE111b at four target sites in T_0_ transgenic rice lines. **(B)** Summary of editing activities induced by rBE111a and rBE111b. **(C)** Frequencies of cytosine editing within the target regions of four target sites in T_0_ transgenic rice lines using rBE111a and rBE111b. **(D)** Proportion of mutation types induced by rBE111a and rBE111b at four target sites. Bi, bi-allelic mutations; Mo, mono-allelic mutations; WT, wild type.

It has been reported that structurally engineered SpRY can recognize atypical NNN PAMs (Walton et al., 2020; Li et al., 2021; Xu et al., 2021b), thus expanding the target scope of ABEs and AKBEs for targeted rice genome editing (Yan et al., 2021; Wu et al., 2023). Thus, to expand the target scope of TadA-based cytosine base editing, CBE constructs were updated by replacing the SpCas9n gene with SpRYn, resulting in pUbi:rBE111a (TadA-CDd–SpRYn–UGI) and pUbi:rBE111b (TadA-E27R/N46L–SpRYn–UGI). Four target sites with atypical PAMs, *OsCOI2-T1*, *OsSPL7*, *OsWx*, and *OsCOI2-T2*, were chosen to test the C-to-T editing activity of the new constructs toward NNN PAMs in transgenic rice. We observed robust activities of rBE111a and rBE111b at the NGC PAM site in *OsCOI2-T1*, achieving 93.05% and 89.58% efficiencies, and at the NCC PAM site in *OsSPL7*, achieving 83.33% and 85.42% efficiencies, respectively (Figure 2A). Compared with rBE111b, rBE111a showed higher editing efficiencies for *OsWx* (21.80% versus 62.50%) and *OsCOI2-T2* (8.33% versus 37.50%) (Figure 2A). C-to-G/A and indel editing events were also detected in the rBE111a- and rBE111b-mediated rice editing populations (Figure 2B). The enhanced base editing frequencies of multiple targeted cytosines across a broader editing window and the proportions of bi-allelic mutations with only base editing also demonstrated that rBE111a outperformed rBE111b (Figures 2C and 2D; Supplemental Figures 13 and 14). Self-targeting of SpRYn-guided rBE111a and rBE111b was also examined; few self-editing events in the T-DNA region in the T_0_ transgenic lines were observed without an obvious correlation between on-target or self-target editing (Supplemental Figures 13 and 14). These results suggest that TadA-based cytosine base editing is compatible with SpRYn, enabling efficient C-to-T base editing by recognizing a highly flexible PAM in rice, and that TadA-CDd has higher activity in the editing window than TadA-E27R/N46L.

### Novel CGBEs derived from TadA achieved C-to-G base editing

**Figure 3:**
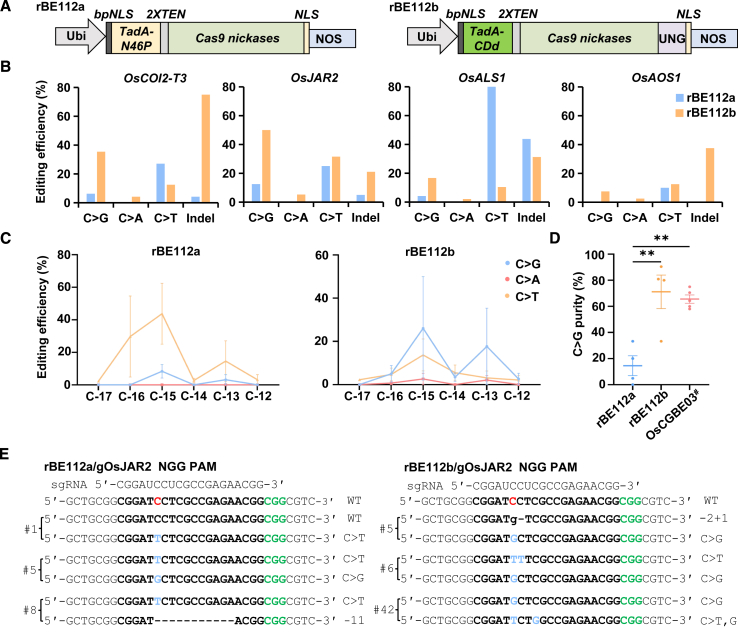
Characterization of TadA-variant-mediated C-to-G base editors with SpCas9n in rice. **(A)** Schematic illustration of TadA-variant-mediated C-to-G base editors with SpCas9n in rice. UNG, uracil DNA glycosylase. **(B)** Comparison of C-to-G editing efficiencies of rBE112a and rBE112b at four target sites in T_0_ transgenic rice lines. **(C)** The activity windows of rBE112a and rBE112b in rice. **(D)** C-to-G conversion purity of rBE112a, rBE110b, and a previously reported C-to-G base editor, OsCGBE03. ^#^The data showing the C-to-G editing purity of OsCGBE03 are cited from Tian et al. (2022). Each dot indicates an individual target site, and the bold lines represent the mean of base editing frequencies. Data are presented as mean ± SD. *P* values were obtained using two-sided Mann–Whitney tests. ∗*P* < 0.05 and ∗∗*P* < 0.01. **(E)** Sequencing results of the rBE112a- or rBE112b-induced *OsJAR2* mutations in T_0_ transgenic rice lines.

CGBEs are new base editing platforms that are capable of C-to-G editing in mammalian and plant cells and C-to-A editing in bacterial cells, further expanding the base editing toolkit for base transversion (Kurt et al., 2021; Tian et al., 2022). The TadA-N46P variant was reported as an unnatural cytosine deaminase and mainly induced C-to-G transversion with a broader editing window than TadA-N46L in HEK293T cells (Chen et al., 2023), meaning that TadA-N46P is more suitable for endogenous gene evolution in plants. To investigate the C-to-G editing capability of TadA-N46P in plants, we established a CGBE system in rice, rBE112a, by fusing TadA-N46P to the N terminus of SpCas9n (Figure 3A). We also constructed another CGBE system, rBE112b, by replacing UGI with OsUNG in rBE110a, given that TadA-CDd exhibits robust cytosine deaminase activity in rice (Figure 3A). Four target sites (*OsCOI2-T3*, *OsJAR2*, *OsALS1*, and *OsAOS1*) were used to test the C-to-G base editing capabilities of rBE112a and rBE112b. In contrast to its C-to-G editing capability in mammalian cells (Chen et al., 2023), the TadA-N46P-mediated base editor exhibited frequent C-to-T editing and poor efficiency of C-to-G editing with an approximately 4-bp activity window (spanning from protospacer positions −16 to −13) in rice (Figures 3B and 3C). Sequencing results showed that frequent C-to-G/A editing events were successfully induced by TadA-CDd- and OsUNG-based rice CGBE rBE112b, and C-to-G editing events were the dominant base editing outcomes (Figure 3B). For the target site *OsJAR2*, the efficiency of C-to-G editing reached 50.00% (Figure 3B). Remarkably, a large number of indel mutations also appeared in rBE112b editing, with efficiencies from 2.08% to 16.67% (Figure 3B). The activity window of C-to-G transversion induced by rBE112b was also approximately 4 bp (spanning from protospacer positions −13 to −16) (Figure 3C; Supplemental Figures 15–18). In terms of base editing purity, the TadA-CDd-based CGBE rBE112b exhibited comparable or slightly higher C-to-G purity than the previously reported representative CGBE OsCGBE03 (Anc689(R33A)–SpCas9n–OsUNG) (Tian et al., 2022) (Figure 3D). Bi-allelic plants with both base conversions (C-to-G/C-to-T) or C-to-G/indel were also frequently detected (Figure 3E), meaning that the frequent indel mutations do not hamper the application of TadA-CDd-based CGBEs, and the by-product indel can be eliminated during gamete production. TadA-CDd-based CGBEs can be further optimized in the future by introduction of a suicide enzyme, HMCES, that was reported to reduce CGBE-initiated double-stranded breaks by shielding the apurinic/apyrimidinic (AP) site (Huang et al., 2024). In another sense, however, the abundant variations caused by TadA-CDd-based CGBEs are more profitable for the directed evolution of target genes in crops. For example, a 6-bp insertion in the coding region of *OsGS1* created by base-editing-mediated artificial evolution conferred glufosinate tolerance in rice (Ren et al., 2023). Together, these results demonstrate that the TadA-CDd-based CGBE enables highly efficient C-to-G editing in the rice genome and is a potential screening platform for direct evolution within crops.

### Development of a dual cytosine and adenine editor derived from the TadA variant

**Figure 4:**
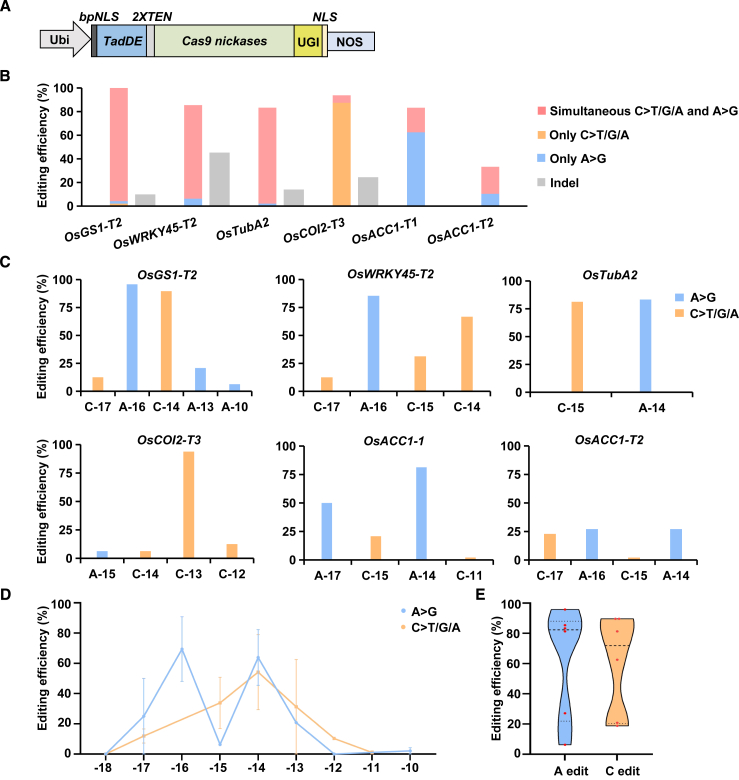
Characterization of a TadDE-mediated dual base editor with SpCas9n in rice. **(A)** Schematic illustration of TadDE-mediated dual base editor with SpCas9n in rice. **(B)** Editing efficiencies of rBE114a at six target sites in T_0_ transgenic rice lines. **(C)** Frequencies of cytosine editing and adenine editing within the target regions of six target sites in T_0_ transgenic rice lines using rBE114a. **(D)** The activity windows of rBE114a in rice. **(E)** Summary of frequencies of cytosine editing or adenine editing introduced by rBE114a.

A dual base editor with the ability to simultaneously convert C-to-T and A-to-G is ideal for artificial evolution of important crop genes. The current dual base editors in plants are established by fusing both cytosine and adenine deaminases to the Cas9 nickase (Li et al., 2020; Xu et al., 2021a). Here, we used the single engineered deaminase TadDE (R26G/V28A/A48R/Y73S/H96N) evolved from TadA8e to establish a new dual base editing system that can simultaneously perform C-to-T and A-to-G transformations. TadDE and UGI were fused to the N and C termini of nicked SpCas9, respectively, resulting in rBE114a (Figure 4A). To examine the base editing activity of rBE114a at rice endogenous genes, we designed sgRNAs targeting six different rice genes, including *OsGS1-T1*, *OsWRKY45-T2*, *OsTubA2*, *OsCOI2-T3*, *OsACC-T1*, and *OsACC-T2*. The results showed that the TadDE- and UGI-constructed rBE114a produced both C-to-T and A-to-G conversions efficiently (Figure 4B). Highly efficient simultaneous C-to-T and A-to-G substitutions were observed at target sites in *OsGS1-T1*, *OsWRKY45-T2*, and *OsTubA2* with frequencies up to 80.0% (Figures 4B and 4C). rBE114a generated higher proportions of transgenic rice lines bearing only a C-to-T or A-to-G substitution than bearing concurrent C-to-T and A-to-G substitutions in target sites of *OsCOI2-T3* and *OsACC-T1*, respectively (Figures 4B and 4C). For the target *OsACC1-T2*, the frequency of simultaneous C-to-T and A-to-G substitutions was only 22.9% (Figures 4B and 4C). These results suggest that the performance of TadDE-mediated C-to-T and A-to-G base editing in rice is locus dependent. The activity window of C-to-T or A-to-G substitution induced by rBE114a was approximately 5 bp, ranging from protospacer positions −17 to −13 (Figure 4D; Supplemental Figures 19–21). This range is narrower than that observed with the previously reported plant dual base editor pDuBE1 (TadA–SpCas9n–LjCDA1L–UGI) (Xu et al., 2021a). Whereas pDuBE1 exhibits higher A-to-G than C-to-T editing activity, rBE114a exhibits very similar A-to-G and C-to-T editing activity (Figure 4E) and is smaller than pDuBE1 in protein size. Moreover, use of the single deaminase TadDE in plant dual base editors can avoid the influences of C- or N-terminal fusion preferences, conformational changes, and competition for the active space and may be more compatible with other Cas proteins. In addition, introducing PAM-flexible SpCas9-NG and SpRY proteins and expanding the coverage of saturation mutations can be used to alleviate the constraints imposed by the narrow editing window (5 bp) of the TadDE-mediated dual base editor for directed evolution. Together, these results indicate that a TadDE-mediated dual base editor is a promising tool for concurrent A-to-G and C-to-T editing in plants and can be used for directed evolution of target genes in crops.

In this study, we investigated the base editing activities of evolved *E. coli* adenine deaminase TadA variants (TadA-CDd, TadA-E27R/N46L, TadA-N46P, and TadDE) in transgenic rice. The data demonstrated that the cytosine base editing activity of TadA-CDd was comparable to or higher than that of natural hyperactive cytosine deaminase hAID∗Δ, and TadDE exhibited robust base editing activity for both cytosine and adenine conversions. Conbining the previously reported TadA9 (Yan et al., 2021), we developed a series of distinct base editors (CBEs, ABEs, CGBEs, AKBEs, and dual base editors) with multifaceted TadA variants to enable almost all 12 types of base conversions and concurrent A·T-to-G·C and C·G-to-T·A conversions (Supplemental Figure 22). Therefore, the combination of TadA variants could greatly facilitate the development of base editors in various plant species by providing high-activity and unitive deaminases, tremendously accelerating basic plant research and crop genetic improvement.

## Methods

### Rice cultivars and growth conditions

The *Geng* rice cultivar Kitaake was used in this study and kept in our laboratory. Rice plants were cultivated in a paddy field under natural conditions of the normal rice growing season, and immature seeds were harvested for rice transformation.

### Plasmid construction

The 651-bp coding regions of TadA-CDd (E27K/V28A/M61I/H96N), TadA-E27R/N46L, TadA-N46P, and TadDE (R26G/V28A/A48R/Y73S/H96N) variants, attached to bpNLS and 2XTEN on both sides, were rice codon optimized and individually synthesized by Tsingke (Beijing, China) (Supplemental Table 1). The synthesized TadA-CDd fragment was amplified with the primer pair bpNLS-F1/CBE1-815-R1 and directly inserted into the backbone of pUC57:TadA-TadA7.10-Cas9(D10A)-fg1 (Yan et al., 2018), which was amplified with the primer pair OsCas9-Fg1-F4/pUC57-bpNLS-R1 using the ClonExpress II One Step Cloning Kit (C112, Vazyme, Nanjing, China), resulting in pUC57:TadA-CDd-Cas9(D10A)-fg1. To construct CBEs with the TadA variants, TadA-CDd-Cas9(D10A)-fg1 released from plasmid pUC57:TadA-CDd-Cas9(D10A)-fg1 by *Bam*HI/*Pae*I digestion and Cas9-fg2-UGI-NLS released from plasmid pUC57-Cas9-fg2-UGI-NLS (Ren et al., 2018) with *Pae*I/*Spe*I were ligated together with the backbone of pUbi:rCas9 (Zhou et al., 2014) digested with *Bam*HI/*Spe*I, resulting in the binary vector pUbi:rBE110a (TadA-CDd–SpCas9n–UGI). Using the same strategy, pUbi:rBE110b (TadA-E27R/N46L–SpCas9n–UGI), pUbi:rBE112a (TadA-N46P–SpCas9n), and pUbi:rBE114a (TadDE–SpCas9n–UGI) were constructed using the synthesized TadA variant fragments. Both rBE110a and rBE110b were under the control of the maize ubiquitin 1 promoter and the nopaline synthase terminator.

The TadA-CDd and TadA-E27R/N46L fragments were fused to the N terminus of SpRYn–UGI amplified from pUC19:rBE66 (Xu et al., 2021b) using an overlapping-extension PCR-based method with the primers listed in Supplemental Table 4, resulting in rBE111a (TadA-CDd–SpRYn–UGI) and rBE111b (TadA-E27R/N46L–SpRYn–UGI) fragments. Finally, rBE111a and rBE111b were cloned into the binary vector pUbi by *Bam*HI/*Spe*I digestion as described above, resulting in pUbi:rBE111a and pUbi:rBE111b.

To generate the TadA-CBEd- and OsUNG-constructed rice CGBEs, the rice codon-optimized and synthesized OsUNG fragment amplified with the primer pair Cas9-UNG-F1/Cas9-UNG-R1 and the approximately 4.1-kb SpCas9n fragment amplified from pUbi:rBE110a with the primer pair Cas9-F/R were cloned into the synthesized TadA-CDd plasmid amplified with the primer pair Cas9-CDd-F1/Cas9-CDd-R1 using the ClonExpress MultiS One Step Cloning Kit (C113, Vazyme), resulting in pUC57:rBE112b (TadA-CDd–SpCas9n–OsUNG). rBE112a was then inserted into the binary vector pUbi by *Bam*HI/*Spe*I digestion as described above, resulting in pUbi:rBE112a.

The sgRNA expression plasmids were constructed and shuttled into appropriate base editor binary vectors (Supplemental Table 2) by LR Clonase (Invitrogen) as described previously (Zhou et al., 2014). For the target site of each gene (Supplemental Table 3), the complementary oligos (Supplemental Table 4) with appropriate 4-bp overhangs were synthesized, annealed, and then inserted into *Bsa*I- or *Btg*ZI-predigested pENTR-sgRNA4.

All PCR amplifications for plasmid construction were performed with Phanta Max Super-Fidelity DNA Polymerase (P505, Vazyme) using the primers listed in Supplemental Table 4.

### *Agrobacterium tumefaciens*–mediated rice transformation

T-DNA transformation plasmids harboring gene-targeting sgRNAs were introduced into *A. tumefaciens* strain EHA105 competent cells by electroporation. The *Agrobacterium*-mediated rice transformation was carried out with immature seed-derived calli following a previously described protocol (Hiei and Komari, 2008; Wang et al., 2022b).

### Genotype and sequence analysis of the transgenic rice lines

Rice genomic DNA was isolated from independent transgenic rice lines using the hexadecyltrimethylammonium bromide method. PCR amplification of the targeted genomic regions was carried out using 2× Rapid Taq Master Mix DNA polymerase (P222, Vazyme) with the specific primers listed in Supplemental Table 4. The PCR products were subjected to Sanger sequencing or deep sequencing using the Hi-TOM platform (Liu et al., 2019) to detect potential mutations.

## Funding

This project was supported by the STI 2030-Major Projects (2023ZD04074), the 10.13039/501100012166National Key Research and Development Program of China (2023YFD1202900), the Nanfan special project of the Chinese Academy of Agricultural Sciences (YBXM2313), the Hainan Seed Industry Laboratory (project of B23CJ0208), and the Agricultural Science and Technology Innovation Program of the 10.13039/501100005196Chinese Academy of Agricultural Sciences.

## Author contributions

H.Z. and B.R. designed the research; M.Y., B.R., Y.K., C.W., and X.W. conducted the experiments; S.L. performed the bioinformatics analysis; X.Z., W.S., and D.Z. supervised the research; M.Y., B.R., and H.Z. wrote the original draft; and all authors participated in discussion and revision of the manuscript.

## Supplemental information

Document S1. Supplemental Figures S1‒S22 and Supplemental Tables S1–S4

Document S2. Article plus supplemental information
