# Supplementary material for: Diverse nucleotide substitutions in rice base editing mediated by novel TadA variants
Source: Plant Commun. 2024 May 8;5(8):100926. doi: 10.1016/j.xplc.2024.100926 (PMC11369719; doi:10.1016/j.xplc.2024.100926)
Supplement: Document S1. Supplemental Figures S1‒S22 and Supplemental Tables S1–S4 [file mmc1.pdf]

Plant Communications, Volume *5*

Supplemental information

Diverse nucleotide substitutions in rice base editing mediated by novel TadA variants

Man Yu, Yongjie Kuang, Chenyang Wang, Xuemei Wu, Shaofang Li, Dawei Zhang, Wenxian Sun, Xueping Zhou, Bin Ren, and Huanbin Zhou

SUPPLEMENTAL INFORMATION

Diverse nucleotide substitutions in rice base editing mediated by novel TadA variants

Man Yu, Yongjie Kuang, Chenyang Wang, Xuemei Wu, Shaofang Li, Dawei Zhang, Wenxian Sun, Xueping Zhou, Bin Ren* & Huanbin Zhou*

CONTENTS

Supplemental Figure 1. Targeted cytosine base editing of *OsCERK1* in rice using rBE9, rBE110a and rBE110b.

Supplemental Figure 2. Targeted cytosine base editing of *OsJAR2* in rice using rBE9, rBE110a and rBE110b.

Supplemental Figure 3. Targeted cytosine base editing of *OsBRI1* in rice using rBE9, rBE110a and rBE110b.

Supplemental Figure 4. Targeted cytosine base editing of *OsBZR1-T1* in rice using rBE9, rBE110a and rBE110b.

Supplemental Figure 5. Targeted cytosine base editing of *OsFLS2* in rice using rBE110a and rBE110b.

Supplemental Figure 6. Targeted cytosine base editing of *OsWRKY45-T1* in rice using rBE110a and rBE110b.

Supplemental Figure 7. Targeted cytosine base editing of *OsGS1-T1* in rice using rBE110a and rBE110b.

Supplemental Figure 8. Targeted cytosine base editing of *OsGS2* in rice using rBE110a and rBE110b.

Supplemental Figure 9. Targeted cytosine base editing of *OsBZR1-T2* in rice using rBE110a and rBE110b.

Supplemental Figure 10. Targeted cytosine base editing of *OsJAR1* in rice using rBE110a and rBE110b.

Supplemental Figure 11. Targeted cytosine base editing of *OsAOS1* in rice using rBE110a and rBE110b.

Supplemental Figure 12. Base editing efficiencies of the rBE110a and rBE110b across the protospacer positions -18~ -12 sites.

Supplemental Figure 13. Targeted cytosine base editing of *OsCOI2-T1* and *OsSPL7* in rice using rBE111a and rBE111b.

Supplemental Figure 14. Targeted cytosine base editing of *OsWx* and *OsCOI2-T2* in rice using rBE111a and rBE111b.

Supplemental Figure 15. Targeted C-to-G base editing of *OsCOI2-T3* in rice using rBE112a and rBE112b.

Supplemental Figure 16. Targeted C-to-G base editing of *OsJAR2* in rice using rBE112a and rBE112b.

Supplemental Figure 17. Targeted C-to-G base editing of *OsALS1* in rice using rBE112a and rBE112b.

Supplemental Figure 18. Targeted C-to-G base editing of *OsAOS1* in rice using rBE112a and rBE112b.

Supplemental Figure 19. Dual cytosine and adenine base editing of *OsGS1-T2* and *OsWRKY45-T2* in rice using rBE114a.

Supplemental Figure 20. Dual cytosine and adenine base editing of *OsTubA2* and *OsCOI2-T3*in rice using rBE114a.

Supplemental Figure 21. Dual cytosine and adenine base editing of *OsACC1-T1* and *OsACC1-T2* in rice using rBE114a.

Supplemental Figure 22. Diagram showing 12 types of site-specific point mutations that can be achieved with available base editors in rice.

Supplemental Table 1. The nucleotide sequences of the rice codon-optimized *TadA-CDd*, *TadA-E27R/N46L*, *TadA-N46P*, *TadA-dual* and *OsUNG* gene fragments.

Supplemental Table 2. Genome editing tools generated and used in this study.

Supplemental Table 3. Rice genes used for targeted base editing in this study.

Supplemental Table 4. List of oligonucleotides in this study.

A rBE9:*OsCERK1*

%	C	C	A	G	T	A	T	A	C	A	T	A	C	A	T	C	G_{-14}	G_{-15}	G_{-16}	A	C	A	T	Indel
A	0.0	0.0	/	0.0	0.0	/	0.0	/	0.0	/	0.0	/	0.0	/	0.0	0.0	2.1	14.6	20.8	/	0.0	/	0.0	4.2
T	0.0	0.0	0.0	0.0	/	0.0	/	0.0	0.0	0.0	/	0.0	0.0	0.0	/	0.0	0.0	0.0	0.0	0.0	0.0	0.0	/	
C	/	/	0.0	0.0	0.0	0.0	0.0	0.0	/	0.0	0.0	0.0	/	0.0	0.0	/	0.0	2.1	0.0	0.0	/	0.0	0.0	
G	0.0	0.0	0.0	/	0.0	0.0	0.0	0.0	0.0	0.0	0.0	0.0	0.0	0.0	0.0	0.0	/	/	/	0.0	0.0	0.0	0.0	

B rBE110a:*OsCERK1*

%	C	C	A	G	T	A	T	A	C	A	T	A	C	A	T	C	G_{-14}	G_{-15}	G_{-16}	A	C	A	T	Indel
A	0.0	0.0	/	0.0	0.0	/	0.0	/	0.0	/	0.0	/	0.0	/	0.0	0.0	85.4	85.4	83.3	/	0.0	/	0.0	10.4
T	0.0	0.0	0.0	0.0	/	0.0	/	0.0	0.0	0.0	/	0.0	0.0	0.0	/	0.0	0.0	0.0	2.1	0.0	0.0	0.0	/	
C	/	/	0.0	0.0	0.0	0.0	0.0	0.0	/	0.0	0.0	0.0	/	0.0	0.0	/	0.0	0.0	2.1	0.0	/	0.0	0.0	
G	0.0	0.0	0.0	/	0.0	0.0	0.0	0.0	0.0	0.0	0.0	0.0	0.0	0.0	0.0	0.0	/	/	/	0.0	0.0	0.0	0.0	

C rBE110b:*OsCERK1*

%	C	C	A	G	T	A	T	A	C	A	T	A	C	A	T	C	G_{-14}	G_{-15}	G_{-16}	A	C	A	T	Indel
A	0.0	0.0	/	0.0	0.0	/	0.0	/	0.0	/	0.0	/	0.0	/	0.0	0.0	81.3	81.3	81.3	/	0.0	/	0.0	2.1
T	0.0	0.0	0.0	0.0	/	0.0	/	0.0	0.0	0.0	/	0.0	0.0	0.0	/	0.0	0.0	0.0	0.0	0.0	0.0	0.0	/	
C	/	/	0.0	0.0	0.0	0.0	0.0	0.0	/	0.0	0.0	0.0	/	0.0	0.0	/	0.0	0.0	0.0	0.0	/	0.0	0.0	
G	0.0	0.0	0.0	/	0.0	0.0	0.0	0.0	0.0	0.0	0.0	0.0	0.0	0.0	0.0	0.0	/	/	/	0.0	0.0	0.0	0.0	

D

rBE9/gOsCERK1 NGG PAM

```
  sgRNA 5′-GUAUACAUACAUCGGGACAU-3′
5′-CTGTTCCAGTATACATACATCGGGACATCAAAT-3′      WT
5′-CTGTTCCAGTATACATACATCGAAACATCAAAT-3′   5/48 10.4%
5′-CTGTTCCAGTATACATACATCGGAACATCAAAT-3′   4/48  8.3%
5′-CTGTTCCAGTATACATACATCAAAACATCAAAT-3′   1/48  2.1%
5′-CTGTTCCAGTATACATACATCGCAACATCAAAT-3′   1/48  2.1%
5′-CTGTTCCAGTATACATACATCGAGACATCAAAT-3′   1/48  2.1%
5′-CTGTTCCAGTATAC-------(-48)--------3′   1/48  2.1%
5′-CTGTTCCAGTATACATACATAA---CATCAAAT-3′   1/48  2.1%
```

E

rBE110a/gOsCERK1 NGG PAM

```
  sgRNA 5′-GUAUACAUACAUCGGGACAU-3′
5′-CTGTTCCAGTATACATACATCGGGACATCAAAT-3′       WT
5′-CTGTTCCAGTATACATACATCAAAACATCAAAT-3′   39/48 72.9%
5′-CTGTTCCAGTATACATACATCAAGACATCAAAT-3′   12/48 25.0%
5′-CTGTTCCAGTATACATACATCGAAACATCAAAT-3′    4/48  8.3%
5′-CTGTTCCAGTATACATACATCAGAACATCAAAT-3′    1/48  2.1%
5′-CTGTTCCAGTATACATACATCAATACATCAAAT-3′    1/48  2.1%
5′-CTGTTCCAGTATACATACATCAGGACATCAAAT-3′    1/48  2.1%
5′-CTGTTCCAGTATACATACATCGAGACATCAAAT-3′    1/48  2.1%
5′-CTGTTCCAGTATACATACATCGGCACATCAAAT-3′    1/48  2.1%
5′-CTGTTCCAGTATACATACATCAG-ACATCAAAT-3′    1/48  2.1%
5′-CTGTTCCAGTATACATACATCaa-ACcTtAAAT-3′    1/48  2.1%
5′-CTGTTCCAGTATACATA--(-7)-ACATCAAAT-3′    1/48  2.1%
5′-CTGTTCCAGTATACATACAT-(-6)-ATCAAAT-3′    1/48  2.1%
5′-CTGTTC---------------(-30)--------3′    1/48  2.1%
5′---------------(-64)---------------3′    1/48  2.1%
```

F

rBE110b/gOsCERK1 NGG PAM

```
  sgRNA 5′-GUAUACAUACAUCGGGACAU-3′
5′-CTGTTCCAGTATACATACATCGGGACATCAAAT-3′      WT
5′-CTGTTCCAGTATACATACATCAAAACATCAAAT-3′  39/48 81.3%
5′-CTGTTCCAGTATACATACATCAAGACATCAAAT-3′  11/48 22.9%
5′-CTGTTCCAGTATACATACATCGAAACATCAAAT-3′   2/48  4.2%
5′-CTGTTCCAGTATACATACATCAGGACATCAAAT-3′   1/48  2.1%
5′-CTGTTCCAGTATACATACATCGAGACATCAAAT-3′   1/48  2.1%
5′-CTGTTCCAGTATACATACATCGAGACATCAAAT-3′   1/48  2.1%
5′-CTGTTCCAGTATACATACAT-----CATCAAAT-3′   1/48  2.1%
```

Supplemental Figure 1. Targeted cytosine base editing of *OsCERK1* in rice using rBE9, rBE110a and rBE10b.

(A, B and C) Frequencies of C•G to T•A conversion across the target region in OsCERK1 in T0 transgenic rice callus lines. The first nucleotide upstream of the PAM is counted as position -1; the detected nucleotide changes are highlighted in red.

(D, E and F) Representative edited alleles and editing efficiency of *OsCERK1* generated by each CRISPR/SpCas9n-based CBE tool in T0 transgenic rice callus lines. The PAM sequences, target bases in the editing window, and detected nucleotide changes are highlighted in green, red, and blue, respectively.

A rBE9:*OsJAR2*

%	C	G	G	A	T	C_{-15}	C_{-14}	T	C_{-12}	G	C	C	G	A	G	A	A	C	G	G	C	G	G	Indel
A	0.0	0.0	0.0	/	0.0	0.0	0.0	0.0	0.0	0.0	0.0	0.0	0.0	/	0.0	/	/	0.0	0.0	0.0	0.0	0.0	0.0	0.0
T	0.0	0.0	0.0	0.0	/	8.3	2.1	/	2.1	0.0	0.0	0.0	0.0	0.0	0.0	0.0	0.0	0.0	0.0	0.0	0.0	0.0	0.0	
C	/	0.0	0.0	0.0	0.0	/	/	0.0	/	0.0	/	/	0.0	0.0	0.0	0.0	0.0	/	0.0	0.0	/	0.0	0.0	
G	0.0	/	/	0.0	0.0	0.0	4.2	0.0	0.0	/	0.0	0.0	/	0.0	/	0.0	0.0	0.0	/	/	0.0	/	/	

B rBE110a:*OsJAR2*

%	C	G	G	A	T	C_{-15}	C_{-14}	T	C_{-12}	G	C	C	G	A	G	A	A	C	G	G	C	G	G	Indel
A	0.0	0.0	0.0	/	0.0	0.0	0.0	0.0	0.0	0.0	0.0	0.0	0.0	/	0.0	/	/	0.0	0.0	0.0	0.0	0.0	0.0	14.6
T	0.0	0.0	0.0	0.0	/	85.4	79.2	/	2.1	0.0	0.0	0.0	0.0	0.0	0.0	0.0	0.0	0.0	0.0	0.0	0.0	0.0	0.0	
C	/	0.0	0.0	0.0	0.0	/	/	0.0	/	0.0	/	/	0.0	0.0	0.0	0.0	0.0	/	0.0	0.0	/	0.0	0.0	
G	0.0	/	/	0.0	0.0	0.0	16.7	0.0	0.0	/	0.0	0.0	/	0.0	/	0.0	0.0	0.0	/	/	0.0	/	/	

C rBE110b:*OsJAR2*

%	C	G	G	A	T	C_{-15}	C_{-14}	T	C	G	C	C	G	A	G	A	A	C	G	G	C	G	G	Indel
A	0.0	0.0	0.0	/	0.0	0.0	0.0	0.0	0.0	0.0	0.0	0.0	0.0	/	0.0	/	/	0.0	0.0	0.0	0.0	0.0	0.0	0.0
T	0.0	0.0	0.0	0.0	/	37.5	6.3	/	0.0	0.0	0.0	0.0	0.0	0.0	0.0	0.0	0.0	0.0	0.0	0.0	0.0	0.0	0.0	
C	/	0.0	0.0	0.0	0.0	/	/	0.0	/	0.0	/	/	0.0	0.0	0.0	0.0	0.0	/	0.0	0.0	/	0.0	0.0	
G	0.0	/	/	0.0	0.0	4.2	0.0	0.0	0.0	/	0.0	0.0	/	0.0	/	0.0	0.0	0.0	/	/	0.0	/	/	

D

rBE9/gOsJAR2 NGG PAM

```
  sgRNA 5′-CGGAUCCUCGCCGAGAACGG-3′
5′-CGCTGCGGCGGATCCTCGCCGAGAACGGCGGCG-3′     WTa
5′-CGCTGCGGCGGATTGTCGCCGAGAACGGCGGCG-3′   2/48 4.2%
5′-CGCTGCGGCGGATTCTCGCCGAGAACGGCGGCG-3′   1/48 2.1%
5′-CGCTGCGGCGGATTTTTGCCGAGAACGGCGGCG-3′   1/48 2.1%
```

E

rBE110a/gOsJAR2 NGG PAM

```
  sgRNA 5′-CGGAUCCUCGCCGAGAACGG-3′
5′-CGCTGCGGCGGATCCTCGCCGAGAACGGCGGCG-3′      WT
5′-CGCTGCGGCGGATTTTCGCCGAGAACGGCGGCG-3′   34/48 70.8%
5′-CGCTGCGGCGGATTGTCGCCGAGAACGGCGGCG-3′    8/48 16.7%
5′-CGCTGCGGCGGATTCTCGCCGAGAACGGCGGCG-3′    8/48 16.7%
5′-CGCTGCGGCGGATTTTTGCCGAGAACGGCGGCG-3′    1/48  2.1%
5′-CGCTGCGGCGGAT--TaGCCGAGAACGGCGGCG-3′    1/48  2.1%
5′-CGCTGCGGCGGATCCTCcGCCGAGAACGGCGGC-3′    1/48  2.1%
5′-CGCTGCGGCGGATCCTC-------ACGGCGGCG-3′    1/48  2.1%
5′-CGCTGCGGCGGAT--(-10)---AACGGCGGCG-3′    1/48  2.1%
5′-CGCTGCGGCGGA----(-14)-----GGCGGCG-3′    1/48  2.1%
5′-CGCTGCGGCGGATTC---(-11)---GGCGGCG-3′    1/48  2.1%
5′-CGCTGCGGCGGAT---(-11)---ACGGCGGCG-3′    1/48  2.1%
```

F

rBE110b/gOsJAR2 NGG PAM

```
  sgRNA 5′-CGGAUCCUCGCCGAGAACGG-3′
5′-CGCTGCGGCGGATCCTCGCCGAGAACGGCGGCG-3′      WT
5′-CGCTGCGGCGGATTCTCGCCGAGAACGGCGGCG-3′   16/48 33.3%
5′-CGCTGCGGCGGATTTTCGCCGAGAACGGCGGCG-3′    2/48  4.2%
5′-CGCTGCGGCGGATGCTCGCCGAGAACGGCGGCG-3′    2/48  4.2%
5′-CGCTGCGGCGGATCTTCGCCGAGAACGGCGGCG-3′    1/48  2.1%
```

Supplemental Figure 2. Targeted cytosine base editing of *OsJAR2* in rice using rBE9, rBE110a and rBE110b.

(A, B and C) Frequencies of nucleotide conversion across the target region in *OsJAR2* in T0 transgenic rice lines. The first nucleotide upstream of the PAM is counted as position -1; the detected nucleotide changes are highlighted in red.

(D, E and F) Representative edited alleles and editing efficiency of *OsJAR2* generated by each CRISPR/SpCas9n-based CBE tool in T0 transgenic rice lines. The PAM sequences, target bases in the editing window, and detected nucleotide changes are highlighted in green, red, and blue, respectively.

A rBE9:*OsBRI1*

%	G	C	C_{-18}	A	T	T	G	C_{-13}	A	G	A	A	T	C_{-7}	T	C	A	C	C	C	T	G	G	Indel
A	0.0	0.0	0.0	/	0.0	0.0	0.0	0.0	/	0.0	/	/	0.0	0.0	0.0	0.0	/	0.0	0.0	0.0	0.0	0.0	0.0	4.2
T	0.0	0.0	2.1	0.0	/	/	0.0	4.2	0.0	0.0	0.0	0.0	/	2.1	/	0.0	0.0	0.0	0.0	0.0	/	0.0	0.0	
C	0.0	/	/	0.0	0.0	0.0	0.0	/	0.0	0.0	0.0	0.0	0.0	/	0.0	/	0.0	/	/	/	0.0	0.0	0.0	
G	/	0.0	0.0	0.0	0.0	0.0	/	0.0	0.0	/	0.0	0.0	0.0	0.0	0.0	0.0	0.0	0.0	0.0	0.0	0.0	/	/	

B rBE110a:*OsBRI1*

%	G	C	C_{-18}	A	T	T	G	C_{-13}	A	G	A	A	T	C	T	C	A	C	C	C	T	G	G	Indel
A	0.0	0.0	0.0	/	0.0	0.0	0.0	0.0	/	0.0	/	/	0.0	0.0	0.0	0.0	/	0.0	0.0	0.0	0.0	0.0	0.0	0.0
T	0.0	0.0	2.1	0.0	/	/	0.0	37.5	0.0	0.0	0.0	0.0	/	0.0	/	0.0	0.0	0.0	0.0	0.0	/	0.0	0.0	
C	0.0	/	/	0.0	0.0	0.0	0.0	/	0.0	0.0	0.0	0.0	0.0	/	0.0	/	0.0	/	/	/	0.0	0.0	0.0	
G	/	0.0	0.0	0.0	0.0	0.0	/	2.1	0.0	/	0.0	0.0	0.0	0.0	0.0	0.0	0.0	0.0	0.0	0.0	0.0	/	/	

C rBE110b:*OsBRI1*

%	G	C	C_{-18}	A	T	T	G	C_{-13}	A	G	A	A	T	C_{-7}	T	C	A	C	C	C	T	G	G	Indel
A	0.0	0.0	0.0	/	0.0	0.0	0.0	0.0	/	0.0	/	/	0.0	0.0	0.0	0.0	/	0.0	0.0	0.0	0.0	0.0	0.0	2.3
T	0.0	0.0	3.4	0.0	/	/	0.0	2.3	0.0	0.0	0.0	0.0	/	1.1	/	0.0	0.0	0.0	0.0	0.0	/	0.0	0.0	
C	0.0	/	/	0.0	0.0	0.0	0.0	/	0.0	0.0	0.0	0.0	0.0	/	0.0	/	0.0	/	/	/	0.0	0.0	0.0	
G	/	0.0	0.0	0.0	0.0	0.0	/	2.3	0.0	/	0.0	0.0	0.0	0.0	0.0	0.0	0.0	0.0	0.0	0.0	0.0	/	/	

D

rBE9/gOsBRI1 NGG PAM

```
  sgRNA 5’-GCCAUUGCAGAAUCUCACCC-3’
5’-TTCGAGAAGCCATTGCAGAATCTCACCCTGGCT-3’   WT
5’-TTCGAGAAGCCATTGTAGAATCTCACCCTGGCT-3’   2/48  4.2%
5’-TTCGAGAAGCCATTGCAGAATTTCACCCTGGCT-3’   1/48  2.1%
5’-TTCGAGAAGCTATTGTAGAATCTCACCCTGGCT-3’   1/48  2.1%
5’-TTCGAGAAGC------AGAATCTCACCCTGGCT-3’   1/48  2.1%
5’-TTCGAGAAGCCATTGCA--------CCCTGGCT-3’   1/48  2.1%
```

E

rBE110a/gOsBRI1 NGG PAM

```
  sgRNA 5’-GCCAUUGCAGAAUCUCACCC-3’
5’-TTCGAGAAGCCATTGCAGAATCTCACCCTGGCT-3’     WT
5’-TTCGAGAAGCCATTGTAGAATCTCACCCTGGCT-3’  18/48  37.5%
5’-TTCGAGAAGCTATTGCAGAATCTCACCCTGGCT-3’   1/48   2.1%
5’-TTCGAGAAGCCATTGGAGAATCTCACCCTGGCT-3’   1/48   2.1%
```

F

rBE110b/gOsBRI1 NGG PAM

```
  sgRNA 5’-GCCAUUGCAGAAUCUCACCC-3’
5’-TTCGAGAAGCCATTGCAGAATCTCACCCTGGCT-3’     WT
5’-TTCGAGAAGCTATTGCAGAATCTCACCCTGGCT-3’    3/88 3.4%
5’-TTCGAGAAGCCATTGGAGAATCTCACCCTGGCT-3’    2/88 2.3%
5’-TTCGAGAAGCCATTGTAGAATCTCACCCTGGCT-3’    2/88 2.3%
5’-TTCGAGAAGCCATTGCAGAATTTCACCCTGGCT-3’    1/88 1.1%
5’-TTCGAGAAGCCATTGCAGAATCTCtcACCCTGG-3’    1/88 1.1%
5’-TTCGAGAAGCCA--caaatggccacaaaTGGCT-3’    1/88 1.1%
```

Supplemental Figure 3. Targeted cytosine base editing of *OsBRI1* in rice using rBE9, rBE110a and rBE110b.

(A, B and C) Frequencies of nucleotide conversion across the target region in *OsBRI1* in T0 transgenic rice lines. The first nucleotide upstream of the PAM is counted as position -1; the detected nucleotide changes are highlighted in red.

(D, E and F) Representative edited alleles and editing efficiency of *OsBRI1* generated by each CRISPR/SpCas9n-based CBE tool in T0 transgenic rice lines. The PAM sequences, target bases in the editing window, and detected nucleotide changes are highlighted in green, red, and blue, respectively.

A rBE110a:*OsBZR1-T1*

%	A	G	C	A	C	C_{-15}	C_{-14}	G	G	A	C	A	C	G	A	T	A	C	C	G	G	A	G	Indel
A	/	0.0	0.0	/	0.0	0.0	0.0	0.0	0.0	/	0.0	/	0.0	0.0	/	0.0	/	0.0	0.0	0.0	0.0	/	0.0	0.0
T	0.0	0.0	0.0	0.0	0.0	27.1	20.8	0.0	0.0	0.0	0.0	0.0	0.0	0.0	0.0	/	0.0	0.0	0.0	0.0	0.0	0.0	0.0	
C	0.0	0.0	/	0.0	/	/	/	0.0	0.0	0.0	/	0.0	/	0.0	0.0	0.0	0.0	/	/	0.0	0.0	0.0	0.0	
G	0.0	/	0.0	0.0	0.0	0.0	0.0	/	/	0.0	0.0	0.0	0.0	/	0.0	0.0	0.0	0.0	0.0	/	/	0.0	/	

B rBE110b:*OsBZR1-T1*

%	A	G	C	A	C_{-16}	C_{-15}	C_{-14}	G	G	A	C	A	C	G	A	T	A	C	C	G	G	A	G	Indel
A	/	0.0	0.0	/	0.0	0.0	0.0	0.0	0.0	/	0.0	/	0.0	0.0	/	0.0	/	0.0	0.0	0.0	0.0	/	0.0	7.1
T	0.0	0.0	0.0	0.0	1.8	1.8	3.6	0.0	0.0	0.0	0.0	0.0	0.0	0.0	0.0	/	0.0	0.0	0.0	0.0	0.0	0.0	0.0	
C	0.0	0.0	/	0.0	/	/	/	0.0	0.0	0.0	/	0.0	/	0.0	0.0	0.0	0.0	/	/	0.0	0.0	0.0	0.0	
G	0.0	/	0.0	0.0	0.0	0.0	0.0	/	/	0.0	0.0	0.0	0.0	/	0.0	0.0	0.0	0.0	0.0	/	/	0.0	/	

C

rBE110a/gOsBZR1-T1 NAG PAM

sgRNA 5′-AGCACCCGGACACGAUACCG-3′

5′-CCGCCTCGA**GCACCCGGACACGATACCGGAG**TG-3′ WT

5′-CCGCCTCGAGCACTTGGACACGATACCGGAGTG-3′ 10/48 20.8%

5′-CCGCCTCGAGCACTCGGACACGATACCGGAGTG-3′ 3/48 6.3%

D

rBE110b/gOsBZR1-T1 NAG PAM

sgRNA 5′-AGCACCCGGACACGAUACCG-3′

5′-CCGCCTCGA**GCACCCGGACACGATACCGGAG**TG-3′ WT

5′-CCGCCTCGAGCATTTGGACACGATACCGGAGTG-3′ 1/56 1.8%

5′-CCGCCTCGAGCACCTGGACACGATACCGGAGTG-3′ 1/56 1.8%

5′-CCGCCTCGAGCACCCGGACAC-----CGGAGTG-3′ 1/56 1.8%

5′--------(-26)-------ACGATACCGGAGTG-3′ 1/56 1.8%

5′--------(-28)-------ACGATACCGGAGTG-3′ 1/56 1.8%

5′--------(-55)----------------------3′ 1/56 1.8%

Supplemental Figure 4. Targeted cytosine base editing of *OsBZR1-T1* in rice using rBE110a and rBE110b.

(A and B) Frequencies of nucleotide conversion across the target region in *OsBZR1-T1* in T0 transgenic rice lines. The first nucleotide upstream of the PAM is counted as position -1; the detected nucleotide changes are highlighted in red.

(C and D) Representative edited alleles and editing efficiency of *OsBZR1-T1* generated by each CRISPR/SpCas9n-based CBE tool in T0 transgenic rice lines. The PAM sequences, target bases in the editing window, and detected nucleotide changes are highlighted in green, red, and blue, respectively.

A rBE110a:*OsFLS2*

%	C	C	C	C	G	T	C	G	T	G	C	A	C	T	G	C	G-14	A	C	G	T	C	A	Indel
A	0.0	0.0	0.0	0.0	0.0	0.0	0.0	0.0	0.0	0.0	0.0	/	0.0	0.0	0.0	0.0	81.3	/	0.0	0.0	0.0	0.0	/	29.2
T	0.0	0.0	0.0	0.0	0.0	/	0.0	0.0	/	0.0	0.0	0.0	0.0	/	0.0	0.0	0.0	0.0	0.0	0.0	/	0.0	0.0	
C	/	/	/	/	0.0	0.0	/	0.0	0.0	0.0	/	0.0	/	0.0	0.0	/	12.5	0.0	/	0.0	0.0	/	0.0	
G	0.0	0.0	0.0	0.0	/	0.0	0.0	/	0.0	/	0.0	0.0	0.0	0.0	/	0.0	/	0.0	0.0	/	0.0	0.0	0.0	

B rBE110b:*OsFLS2*

%	C	C	C	C	G	T	C	G	T	G	C	A	C	T	G	C	G-14	A	C	G	T	C	A	Indel
A	0.0	0.0	0.0	0.0	0.0	0.0	0.0	0.0	0.0	0.0	0.0	/	0.0	0.0	0.0	0.0	50.0	/	0.0	0.0	0.0	0.0	/	16.7
T	0.0	0.0	0.0	0.0	0.0	/	0.0	0.0	/	0.0	0.0	0.0	0.0	/	0.0	0.0	0.0	0.0	0.0	0.0	/	0.0	0.0	
C	/	/	/	/	0.0	0.0	/	0.0	0.0	0.0	/	0.0	/	0.0	0.0	/	16.7	0.0	/	0.0	0.0	/	0.0	
G	0.0	0.0	0.0	0.0	/	0.0	0.0	/	0.0	/	0.0	0.0	0.0	0.0	/	0.0	/	0.0	0.0	/	0.0	0.0	0.0	

C

rBE110a/gOsFLS2 NGG PAM

```
  sgRNA 5'-CGUCGUGCACUGCGACGUCA-3'
5'-GACTTCCCCGTCGTGCACTGCGACGTCAAGCCG-3'      WT
5'-GACTTCCCCGTCGTGCACTGCAACGTCAAGCCG-3'   39/48 81.3%
5'-GACTTCCCCGTCGTGCACTGCCACGTCAAGCCG-3'    6/48 12.5%
5'-GACTTCCCCGTCGTGCACTGC-ACGTCAAGCCG-3'    4/48  8.3%
5'-GACTTCCCCGT---(-10)--GACGTCAAGCCG-3'    1/48  2.1%
5'-GACTTCCCCGTCGTGCACTGCGcactgcgACGT-3'    1/48  2.1%
5'-GACTTCCCCGTCGTGCACT-------CAAGCCG-3'    1/48  2.1%
5'-GACTTCCCCGTC------(-15)----AAGCCG-3'    1/48  2.1%
5'-GACTTCCCCGTCGTGCACT---ACGTCAAGCCG-3'    1/48  2.1%
5'-GACTTCCCCGTCG--(-9)---ACGTCAAGCCG-3'    1/48  2.1%
5'-GACTTCCCCG-(-6)-ACTGCGACGTCAAGCCG-3'    1/48  2.1%
5'-GACTTCCCCGTCGTGCACTG-GACGTCAAGCCG-3'    1/48  2.1%
5'-GACTTCCCCGTCGTGC-----GACGTCAAGCCG-3'    1/48  2.1%
5'-GACTTCCCCGTCGTGCACTGCaacgcGACGCCG-3'    1/48  2.1%
5'-GACTTCCCCGTC--------(-23)---------3'    1/48  2.1%
5'-GACTTCCCCGTCGTGCACTGCG-CGTCAAGCCG-3'    1/48  2.1%
```

D

rBE110b/gOsFLS2 NGG PAM

```
  sgRNA 5'-CGUCGUGCACUGCGACGUCA-3'
5'-GACTTCCCCGTCGTGCACTGCGACGTCAAGCCG-3'      WT
5'-GACTTCCCCGTCGTGCACTGCAACGTCAAGCCG-3'   24/48 50.0%
5'-GACTTCCCCGTCGTGCACTGCCACGTCAAGCCG-3'    8/48 16.6%
5'-GACTTCCCCGTCGTGCACTGCaacacGACGCCG-3'    1/48  2.1%
5'-GACTTCCCCGT---(-11)---ACGTCAAGCCG-3'    1/48  2.1%
5'-GACTTCCCCGTCGTGCACTGCGtgcacgAGCCG-3'    1/48  2.1%
5'-GACTTCCCCGTCGTGCACTGC------AAGCCG-3'    1/48  2.1%
5'-GACTTCCCCGTCGT---(-12)----CAAGCCG-3'    1/48  2.1%
5'-GACTTCCCCGTCGTGCACTGCaAaaCGTCGCCG-3'    3/48  6.3%
```

Supplemental Figure 5. Targeted cytosine base editing of *OsFLS2* in rice using rBE110a and rBE110b.
(A and B) Frequencies of nucleotide conversion across the target region in *OsFLS2* in T0 transgenic rice lines. The first nucleotide upstream of the PAM is counted as position -1; the detected nucleotide changes are highlighted in red.
(C and D) Representative edited alleles and editing efficiency of *OsFLS2* generated by each CRISPR/SpCas9n-based CBE tool in T0 transgenic rice lines. The PAM sequences, target bases in the editing window, and detected nucleotide changes are highlighted in green, red, and blue, respectively.

A rBE110a:*OsWRKY45-T1*

%	T	G	G	A	G	C$_{-15}$	T	A	C$_{-12}$	G	A	C	G	C	C	G	T	C	G	C	C	G	G	Indel
A	0.0	0.0	0.0	/	0.0	0.0	0.0	/	0.0	0.0	/	0.0	0.0	0.0	0.0	0.0	0.0	0.0	0.0	0.0	0.0	0.0	0.0	18.8
T	/	0.0	0.0	0.0	0.0	91.7	/	0.0	4.2	0.0	0.0	0.0	0.0	0.0	0.0	0.0	/	0.0	0.0	0.0	0.0	0.0	0.0	
C	0.0	0.0	0.0	0.0	0.0	/	0.0	0.0	/	0.0	0.0	/	0.0	/	/	0.0	0.0	/	0.0	/	/	0.0	0.0	
G	0.0	/	/	0.0	/	14.6	0.0	0.0	4.2	/	0.0	0.0	/	0.0	0.0	/	0.0	0.0	/	0.0	0.0	/	/	

B rBE110b:*OsWRKY45-T1*

%	T	G	G	A	G	C$_{-15}$	T	A	C	G	A	C	G	C	C	G	T	C	G	C	C	G	G	Indel
A	0.0	0.0	0.0	/	0.0	0.0	0.0	/	0.0	0.0	/	0.0	0.0	0.0	0.0	0.0	0.0	0.0	0.0	0.0	0.0	0.0	0.0	6.3
T	/	0.0	0.0	0.0	0.0	89.6	/	0.0	0.0	0.0	0.0	0.0	0.0	0.0	0.0	0.0	/	0.0	0.0	0.0	0.0	0.0	0.0	
C	0.0	0.0	0.0	0.0	0.0	/	0.0	0.0	/	0.0	0.0	/	0.0	/	/	0.0	0.0	/	0.0	/	/	0.0	0.0	
G	0.0	/	/	0.0	/	10.4	0.0	0.0	0.0	/	0.0	0.0	/	0.0	0.0	/	0.0	0.0	/	0.0	0.0	/	/	

C

rBE110a/gOsWRKY45-T1 NGG PAM

```
  sgRNA 5'-TGGAGCUACGACGCCGUCGC-3'
5'-CCCTGCACTGGAGCTACGACGCCGTCGCCGGCA-3'      WT
5'-CCCTGCACTGGAGTTACGACGCCGTCGCCGGCA-3'   44/48 83.3%
5'-CCCTGCACTGGAGGTACGACGCCGTCGCCGGCA-3'    7/48 14.6%
5'-CCCTGCACTGGAGTTATGACGCCGTCGCCGGCA-3'    2/48  4.2%
5'-CCCTGCACTGGAGTTAGGACGCCGTCGCCGGCA-3'    2/48  4.2%
5'-CCCTGCACTGGAGCTACGACGCCGccgTCGCCG-3'    2/48  4.2%
5'-CCCTGCACTGGAG--(-10)---GTCGCCGGCA-3'    1/48  2.1%
5'-CCCTGCACTGGAG---(-13)-----GCCGGCA-3'    1/48  2.1%
5'-CCCTGCA----(-17)--------TCGCCGGCA-3'    1/48  2.1%
5'-CCCTGCACTGGAGCTACGACGCCacgccGTCGA-3'    1/48  2.1%
5'-CCCTGCACTGGAGCTACGACGCCGTCcgtcGCA-3'    1/48  2.1%
5'-CCCTGCACTGGAGCTACG--(-9)---CCGGCA-3'    1/48  2.1%
5'-CCCTGCACTGGAGCTACGACGCtacgacgcCGT-3'    1/48  2.1%
```

D

rBE110b/gOsWRKY45-T1 NGG PAM

```
  sgRNA 5'-TGGAGCUACGACGCCGUCGC-3'
5'-CCCTGCACTGGAGCTACGACGCCGTCGCCGGCA-3'      WT
5'-CCCTGCACTGGAGTTACGACGCCGTCGCCGGCA-3'   43/48 89.6%
5'-CCCTGCACTGGAGGTACGACGCCGTCGCCGGCA-3'    5/48 10.4%
5'-CCCTGCACTGGAGCTACGAC-CCGTCGCCGGCA-3'    1/48  2.1%
5'-CCCTGCACTGGAGCTACGACGCC--CGCCGGCA-3'    1/48  2.1%
5'-CCCTGCACTGGAGCTACGACGCCGTacgacgcc-3'    1/48  2.1%
```

Supplemental Figure 6. Targeted cytosine base editing of *OsWRKY45-T1* in rice using rBE110a and rBE110b.

(A and B) Frequencies of nucleotide conversion across the target region in *OsWRKY45-T1* in T0 transgenic rice lines. The first nucleotide upstream of the PAM is counted as position -1; the detected nucleotide changes are highlighted in red.

(C and D) Representative edited alleles and editing efficiency of *OsWRKY45-T1* generated by each CRISPR/SpCas9n-based CBE tool in T0 transgenic rice lines. The PAM sequences, target bases in the editing window, and detected nucleotide changes are highlighted in green, red, and blue, respectively.

A **rBE110a:*OsGS1-T1***

%	C	C	G	G	C	T	C	A	C	C	G_{-8}	G	C	A	G_{-12}	G_{-13}	C	A	C	G	A	G	A	Indel
A	0.0	0.0	0.0	0.0	0.0	0.0	0.0	/	0.0	0.0	4.2	0.0	0.0	/	18.8	6.3	0.0	/	0.0	0.0	/	0.0	/	0.0
T	0.0	0.0	0.0	0.0	0.0	/	0.0	0.0	0.0	0.0	0.0	0.0	0.0	0.0	0.0	0.0	0.0	0.0	0.0	0.0	0.0	0.0	0.0	
C	/	/	0.0	0.0	/	0.0	/	0.0	/	/	0.0	0.0	/	0.0	0.0	0.0	/	0.0	/	0.0	0.0	0.0	0.0	
G	0.0	0.0	/	/	0.0	0.0	0.0	0.0	0.0	0.0	/	/	0.0	0.0	/	/	0.0	0.0	0.0	/	0.0	/	0.0	

B **rBE110b:*OsGS1-T1***

%	C	C	G	G	C	T	C	A	C	C	G	G	C	A	G_{-12}	G	C	A	C	G	A	G	A	Indel
A	0.0	0.0	0.0	0.0	0.0	0.0	0.0	/	0.0	0.0	0.0	0.0	0.0	/	2.1	0.0	0.0	/	0.0	0.0	/	0.0	/	0.0
T	0.0	0.0	0.0	0.0	0.0	/	0.0	0.0	0.0	0.0	0.0	0.0	0.0	0.0	0.0	0.0	0.0	0.0	0.0	0.0	0.0	0.0	0.0	
C	/	/	0.0	0.0	/	0.0	/	0.0	/	/	0.0	0.0	/	0.0	0.0	0.0	/	0.0	/	0.0	0.0	0.0	0.0	
G	0.0	0.0	/	/	0.0	0.0	0.0	0.0	0.0	0.0	/	/	0.0	0.0	/	/	0.0	0.0	0.0	/	0.0	/	0.0	

C

rBE110a/gOsGS1-T1 **NGG PAM**

```
  sgRNA 5'-GCUCACCGGCAGGCACGAGA-3'
5'-GAGCGCCGGCTCACCGGCAGGCACGAGACCGCC-3'     WT
5'-GAGCGCCGGCTCACCGGCAAGCACGAGACCGCC-3'    9/48  18.8%
5'-GAGCGCCGGCTCACCAGCAGGCACGAGACCGCC-3'    2/48   4.2%
5'-GAGCGCCGGCTCACCGGCAGACACGAGACCGCC-3'    3/48   6.3%
```

D

rBE110b/gOsGS1-T1 **NGG PAM**

```
  sgRNA 5'-GCUCACCGGCAGGCACGAGA-3'
5'-GAGCGCCGGCTCACCGGCAGGCACGAGACCGCC-3'      WT
5'-GAGCGCCGGCTCACCGGCAAGCACGAGACCGCC-3'     1/48  2.1%
```

Supplemental Figure 7. Targeted cytosine base editing of *OsGS1-T1* in rice using rBE110a and rBE110b.

(A and B) Frequencies of nucleotide conversion across the target region in *OsGS1-T1* in T0 transgenic rice lines. The first nucleotide upstream of the PAM is counted as position -1; the detected nucleotide changes are highlighted in red.

(C and D) Representative edited alleles and editing efficiency of *OsGS1-T1* generated by each CRISPR/SpCas9n-based CBE tool in T0 transgenic rice lines. The PAM sequences, target bases in the editing window, and detected nucleotide changes are highlighted in green, red, and blue, respectively.

A rBE110a:*OsGS2*

%	C	T	G	C	A	C	A	A	G	T	A	T	T	C	A	G	T	G_{-15}	A	T	C	C	A	Indel
A	0.0	0.0	0.0	0.0	/	0.0	/	/	0.0	0.0	/	0.0	0.0	0.0	/	0.0	0.0	56.3	/	0.0	0.0	0.0	/	6.3
T	0.0	/	0.0	0.0	0.0	0.0	0.0	0.0	0.0	/	0.0	/	/	0.0	0.0	0.0	/	0.0	0.0	/	0.0	0.0	0.0	
C	/	0.0	0.0	/	0.0	/	0.0	0.0	0.0	0.0	0.0	0.0	0.0	/	0.0	0.0	0.0	0.0	0.0	0.0	/	/	0.0	
G	0.0	0.0	/	0.0	0.0	0.0	0.0	0.0	/	0.0	0.0	0.0	0.0	0.0	0.0	/	0.0	/	0.0	0.0	0.0	0.0	0.0	

B rBE110b:*OsGS2*

%	C	T	G	C	A	C	A	A	G	T	A	T	T	C	A	G	T	G_{-15}	A	T	C	C	A	Indel
A	0.0	0.0	0.0	0.0	/	0.0	/	/	0.0	0.0	/	0.0	0.0	0.0	/	0.0	0.0	52.1	/	0.0	0.0	0.0	/	2.1
T	0.0	/	0.0	0.0	0.0	0.0	0.0	0.0	0.0	/	0.0	/	/	0.0	0.0	0.0	/	0.0	0.0	/	0.0	0.0	0.0	
C	/	0.0	0.0	/	0.0	/	0.0	0.0	0.0	0.0	0.0	0.0	0.0	/	0.0	0.0	0.0	2.1	0.0	0.0	/	/	0.0	
G	0.0	0.0	/	0.0	0.0	0.0	0.0	0.0	/	0.0	0.0	0.0	0.0	0.0	0.0	/	0.0	/	0.0	0.0	0.0	0.0	0.0	

C

rBE110a/GS2 NAG PAM

```
  sgRNA 5'-CACAAGUAUUCAGUGAUCCA-3'
5'-CAGGGCTGCACAAGTATTCAGTGATCCAAAGGT-3'      WT
5'-CAGGGCTGCACAAGTATTCAGTAATCCAAAGGT-3'   27/48  56.3%
5'-----------(-29)--------------AGGT-3'    1/48   2.1%
5'-CAGGGCTGCAC--(-9)---GTGATCCAAAGGT-3'    1/48   2.1%
5'-CAGGGCTGCACAAGTAT--(-8)--CCAAAGGT-3'    1/48   2.1%
```

D

rBE110b/GS2 NAG PAM

```
  sgRNA 5'-CACAAGUAUUCAGUGAUCCA-3'
5'-CAGGGCTGCACAAGTATTCAGTGATCCAAAGGT-3'      WT
5'-CAGGGCTGCACAAGTATTCAGTAATCCAAAGGT-3'   25/48  52.1%
5'-CAGGGCTGCACAAGTATTCAGTCATCCAAAGGT-3'    1/48   2.1%
5'-------------(-43)---------AAAGGTT-3'    1/48   2.1%
```

Supplemental Figure 8. Targeted cytosine base editing of *OsGS2* in rice using rBE110a and rBE110b. **(A and B)** Frequencies of nucleotide conversion across the target region in *OsGS2* in T0 transgenic rice lines. The first nucleotide upstream of the PAM is counted as position -1; the detected nucleotide changes are highlighted in red.

(C and D) Representative edited alleles and editing efficiency of *OsGS2* generated by each CRISPR/SpCas9n-based CBE tool in T0 transgenic rice lines. The PAM sequences, target bases in the editing window, and detected nucleotide changes are highlighted in green, red, and blue, respectively.

A **rBE110a:*OsBZR1-T2***

%	A	C	C	T	A	C_{-15}	A	A	C_{-12}	C	T	C	G	T	C	A	A	C	C	C	G	G	G	Indel
A	/	0.0	0.0	0.0	/	0.0	/	/	0.0	0.0	0.0	0.0	0.0	0.0	0.0	/	/	0.0	0.0	0.0	0.0	0.0	0.0	0.0
T	0.0	0.0	0.0	/	0.0	4.2	0.0	0.0	2.1	0.0	/	0.0	0.0	/	0.0	0.0	0.0	0.0	0.0	0.0	0.0	0.0	0.0	
C	0.0	/	/	0.0	0.0	/	0.0	0.0	/	/	0.0	/	0.0	0.0	/	0.0	0.0	/	/	/	0.0	0.0	0.0	
G	0.0	0.0	0.0	0.0	0.0	0.0	0.0	0.0	0.0	0.0	0.0	0.0	/	0.0	0.0	0.0	0.0	0.0	0.0	0.0	/	/	/	

B

rBE110a/gOsBZR1-T2 NGG PAM

sgRNA 5′-ACCUACAACCUCGUCAACCC-3′

5′-CGTCGCCC**ACCTACAACCTCGTCAACCCGGG**CG-3′ WT

5′-CGTCGCCCACCTATAACCTCGTCAACCCGGGCG-3′ 2/48 4.2%

5′-CGTCGCCCACCTACAATCTCGTCAACCCGGGCG-3′ 1/48 2.1%

Supplemental Figure 9. Targeted cytosine base editing of *OsBZR1-T2* in rice using rBE110a and rBE110b.

(A) Frequencies of nucleotide conversion across the target region in *OsBZR1-T2* in T0 transgenic rice lines. The first nucleotide upstream of the PAM is counted as position -1; the detected nucleotide changes are highlighted in red.

(B) Representative edited alleles and editing efficiency of *OsBZR1-T2* generated by each CRISPR/SpCas9n-based CBE tool in T0 transgenic rice lines. The PAM sequences, target bases in the editing window, and detected nucleotide changes are highlighted in green, red, and blue, respectively.

A rBE110a:*OsJAR1*

%	C	C	T	G	C	A	T	C	C	C	A	T	T	G	T	G	T	G_{-15}	T	G_{-17}	C	A	C	Indel
A	0.0	0.0	0.0	0.0	0.0	/	0.0	0.0	0.0	0.0	/	0.0	0.0	0.0	0.0	0.0	0.0	43.8	0.0	2.1	0.0	/	0.0	6.3
T	0.0	0.0	/	0.0	0.0	0.0	/	0.0	0.0	0.0	0.0	/	/	0.0	/	0.0	/	0.0	/	0.0	0.0	0.0	0.0	
C	/	/	0.0	0.0	/	0.0	0.0	/	/	/	0.0	0.0	0.0	0.0	0.0	0.0	0.0	2.1	0.0	0.0	/	0.0	/	
G	0.0	0.0	0.0	/	0.0	0.0	0.0	0.0	0.0	0.0	0.0	0.0	0.0	/	0.0	/	0.0	/	0.0	/	0.0	0.0	0.0	

B rBE110b:*OsJAR1*

%	C	C	T	G	C	A	T	C	C	C	A	T	T	G	T	G	T	G_{-15}	T	G	C	A	C	Indel
A	0.0	0.0	0.0	0.0	0.0	/	0.0	0.0	0.0	0.0	/	0.0	0.0	0.0	0.0	0.0	0.0	20.8	0.0	0.0	0.0	/	0.0	2.1
T	0.0	0.0	/	0.0	0.0	0.0	/	0.0	0.0	0.0	0.0	/	/	0.0	/	0.0	/	0.0	/	0.0	0.0	0.0	0.0	
C	/	/	0.0	0.0	/	0.0	0.0	/	/	/	0.0	0.0	0.0	0.0	0.0	0.0	0.0	0.0	0.0	0.0	/	0.0	/	
G	0.0	0.0	0.0	/	0.0	0.0	0.0	0.0	0.0	0.0	0.0	0.0	0.0	/	0.0	/	0.0	/	0.0	/	0.0	0.0	0.0	

C

rBE110a/gOsJAR1 NGG PAM

```
  sgRNA 5'-GCAUCCCAUUGUGUGUGCAC-3'
5'-CAAATCCTGCATCCCATTGTGTGTGCACAATG-3'    WT
5'-CAAATCCTGCATCCCATTGTGTATGCACAATG-3'   20/48 41.7%
5'-CAAATCCTGCATCCCATTGTGTCTGCACAATG-3'    1/48  2.1%
5'-CAAATCCTGCATCCCATTGTGTATACACAATG-3'    1/48  2.1%
5'-CAAATCCTGCATA---(-11)---GCACAATG-3'    1/48  2.1%
5'-CAAATCCTGCAT---(-10)--GTGCACAATG-3'    1/48  2.1%
5'-CAAATCCTGCATCCCATTGTGTcccattgtgt-3'    1/48  2.1%
```

D

rBE110b/gOsJAR1 NGG PAM

```
  sgRNA 5'-GCAUCCCAUUGUGUGUGCAC-3'
5'-CAAATCCTGCATCCCATTGTGTGTGCACAATG-3'     WT
5'-CAAATCCTGCATCCCATTGTGTATGCACAATG-3'   10/48 20.83%
5'-CAAATCCTGCATCCCATTGTGT-TGCACAATG-3'    1/48   2.1%
```

Supplemental Figure 10. Targeted cytosine base editing of *OsJAR1* in rice using rBE110a and rBE110b.

(A and B) Frequencies of nucleotide conversion across the target region in *OsJAR1* in T0 transgenic rice lines. The first nucleotide upstream of the PAM is counted as position -1; the detected nucleotide changes are highlighted in red.

(C and D) Representative edited alleles and editing efficiency of *OsJAR1* generated by each CRISPR/SpCas9n-based CBE tool in T0 transgenic rice lines. The PAM sequences, target bases in the editing window, and detected nucleotide changes are highlighted in green, red, and blue, respectively.

A rBE110a:*OsAOS1*

%	C	G	G	C-17	C-16	G	C-14	G	A	C	G	G	C	T	T	C	T	T	C	G	C	G	G	Indel
A	0.0	0.0	0.0	0.0	0.0	0.0	0.0	0.0	/	0.0	0.0	0.0	0.0	0.0	0.0	0.0	0.0	0.0	0.0	0.0	0.0	0.0	0.0	18.8
T	0.0	0.0	0.0	10.4	87.5	0.0	77.1	0.0	0.0	0.0	0.0	0.0	0.0	/	/	0.0	/	/	0.0	0.0	0.0	0.0	0.0	
C	/	0.0	0.0	/	/	0.0	/	0.0	0.0	/	0.0	0.0	/	0.0	0.0	/	0.0	0.0	/	0.0	/	0.0	0.0	
G	0.0	/	/	0.0	0.0	/	2.1	/	0.0	0.0	/	/	0.0	0.0	0.0	0.0	0.0	0.0	0.0	/	0.0	/	/	

B rBE110b:*OsAOS1*

%	C	G	G	C-17	C-16	G	C-14	G	A	C	G	G	C	T	T	C	T	T	C	G	C	G	G	Indel
A	0.0	0.0	0.0	0.0	0.0	0.0	0.0	0.0	/	0.0	0.0	0.0	0.0	0.0	0.0	0.0	0.0	0.0	0.0	0.0	0.0	0.0	0.0	4.2
T	0.0	0.0	0.0	2.1	89.6	0.0	87.5	0.0	0.0	0.0	0.0	0.0	0.0	/	/	0.0	/	/	0.0	0.0	0.0	0.0	0.0	
C	/	0.0	0.0	/	/	0.0	/	0.0	0.0	/	0.0	0.0	/	0.0	0.0	/	0.0	0.0	/	0.0	/	0.0	0.0	
G	0.0	/	/	0.0	0.0	/	0.0	/	0.0	0.0	/	/	0.0	0.0	0.0	0.0	0.0	0.0	0.0	/	0.0	/	/	

C

rBE110a/gOsAOS1 NGG PAM

```
  sgRNA 5′-GGCCGCGACGGCUUCUUCG-3′
5′-GCCCGGGCGGCCGCGACGGCTTCTTCGCGGCG-3′      WT
5′-GCCCGGGCGGCTGTGACGGCTTCTTCGCGGCG-3′    30/48 62.5%
5′-GCCCGGGCGGCTGCGACGGCTTCTTCGCGGCG-3′    25/48 52.1%
5′-GCCCGGGCGGCCGTGACGGCTTCTTCGCGGCG-3′    13/48 27.1%
5′-GCCCGGGCGGTTGCGACGGCTTCTTCGCGGCG-3′     2/48 4.2%
5′-GCCCGGGCGGTTGTGACGGCTTCTTCGCGGCG-3′     2/48 4.2%
5′-GCCCGGGCGGTCGTGACGGCTTCTTCGCGGCG-3′     1/48 2.1%
5′-GCCCGGGCGGCCGGGACGGCTTCTTCGCGGCG-3′     1/48 2.1%
5′-GC----(-18)---------TTCTTCGCGGCG-3′     2/48 4.2%
5′-GCCCGGGCGG-----ACGGCTTCTTCGCGGCG-3′     1/48 2.1%
5′-GCCC-----(-20)----------TCGCGGCG-3′     1/48 2.1%
5′-GCCCGGGCGGCCG--ACGGCTTCTTCGCGGCG-3′     1/48 2.1%
5′-GCCCGGGC-----(-15)-----TTCGCGGCG-3′     1/48 2.1%
5′-GCCCGGGCGGCCGCGACGacgGCTTCTTCGCG-3′     1/48 2.1%
5′-GCCCGGGCGGtgG-GACGGCTTCTTCGCGGCG-3′     1/48 2.1%
5′-GCCCGGGCGGC----(-13)----TCGCGGCG-3′     1/48 2.1%
5′-GCCCGGGCGGCCG---(-11)---TCGCGGCG-3′     1/48 2.1%
```

D

rBE110b/gOsAOS1 NGG PAM

```
  sgRNA 5′-GGCCGCGACGGCUUCUUCG-3′
5′-GCCCGGGCGGCCGCGACGGCTTCTTCGCGGCG-3′      WT
5′-GCCCGGGCGGCTGTGACGGCTTCTTCGCGGCG-3′    42/48 87.5%
5′-GCCCGGGCGGCTGCGACGGCTTCTTCGCGGCG-3′    29/48 60.4%
5′-GCCCGGGCGGCCGTGACGGCTTCTTCGCGGCG-3′    10/48 20.8%
5′-GCCCGGGCGGTTGCGACGGCTTCTTCGCGGCG-3′     1/48 2.1%
5′-GC----(-18)---------TTCTTCGCGGCG-3′     2/48 4.2%
```

Supplemental Figure 11. Targeted cytosine base editing of *OsAOS1* in rice using rBE110a and rBE110b.

(A and B) Frequencies of nucleotide conversion across the target region in *OsAOS1* in T0 transgenic rice lines. The first nucleotide upstream of the PAM is counted as position -1; the detected nucleotide changes are highlighted in red.

(C and D) Representative edited alleles and editing efficiency of *OsAOS1* generated by each CRISPR/SpCas9n-based CBE tool in T0 transgenic rice lines. The PAM sequences, target bases in the editing window, and detected nucleotide changes are highlighted in green, red, and blue, respectively.

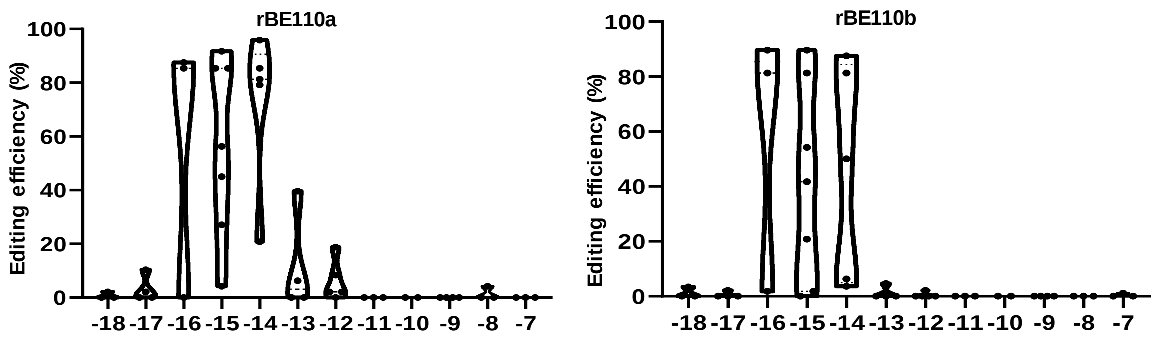

Supplemental Figure 12. Base editing efficiencies of the rBE110a and rBE110b across the protospacer positions -18~ -7 sites.

A rBE111a:*OsCOI2-T1*

%	G	C	C	G	C	C	A	C	T	G	G	T	G-10	C	A	G-13	G-14	G-15	T	C	G-18	A	C	Indel	self-editing
A	0.0	0.0	0.0	0.0	0.0	0.0	/	0.0	0.0	0.0	0.0	0.0	2.8	0.0	/	76.4	88.9	81.9	0.0	0.0	4.2	/	0.0	33.3	2.1
T	0.0	0.0	0.0	0.0	0.0	0.0	0.0	0.0	/	0.0	0.0	/	0.0	0.0	0.0	2.8	0.0	0.0	/	0.0	0.0	0.0	0.0		
C	0.0	/	/	0.0	/	/	0.0	/	0.0	0.0	0.0	0.0	0.0	/	0.0	5.6	5.6	0.0	0.0	/	0.0	0.0	/		
G	/	0.0	0.0	/	0.0	0.0	0.0	0.0	0.0	/	/	0.0	/	0.0	0.0	/	/	/	0.0	0.0	/	0.0	0.0		

rBE111b:*OsCOI2-T1*

%	G	C	C	G	C	C	A	C	T	G	G	T	G	C	A	G-13	G-14	G-15	T	C	G	A	C	Indel	self-editing
A	0.0	0.0	0.0	0.0	0.0	0.0	/	0.0	0.0	0.0	0.0	0.0	0.0	0.0	/	18.8	85.4	64.6	0.0	0.0	0.0	/	0.0	27.1	0.0
T	0.0	0.0	0.0	0.0	0.0	0.0	0.0	0.0	/	0.0	0.0	/	0.0	0.0	0.0	0.0	0.0	0.0	/	0.0	0.0	0.0	0.0		
C	0.0	/	/	0.0	/	/	0.0	/	0.0	0.0	0.0	0.0	0.0	/	0.0	0.0	4.7	0.0	0.0	/	0.0	0.0	/		
G	/	0.0	0.0	/	0.0	0.0	0.0	0.0	0.0	/	/	0.0	/	0.0	0.0	/	/	/	0.0	0.0	/	0.0	0.0		

B rBE111a:*OsSPL7*

%	T	G	C	A	C-16	C-15	T	C-13	C-12	G	A	C	G	C	G	T	G	G	A	C	G	C	C	Indel	self-editing
A	0.0	0.0	0.0	/	0.0	0.0	0.0	0.0	0.0	0.0	/	0.0	0.0	0.0	0.0	0.0	0.0	0.0	/	0.0	0.0	0.0	0.0	3.1	25.0
T	/	0.0	0.0	0.0	16.7	83.3	/	70.8	16.7	0.0	0.0	0.0	0.0	0.0	0.0	/	0.0	0.0	0.0	0.0	0.0	0.0	0.0		
C	0.0	0.0	/	0.0	/	/	0.0	/	/	0.0	0.0	/	0.0	/	0.0	0.0	0.0	0.0	0.0	/	0.0	/	/		
G	0.0	/	0.0	0.0	0.0	0.0	0.0	0.0	0.0	/	0.0	0.0	/	0.0	/	0.0	/	/	0.0	0.0	/	0.0	0.0		

rBE111b:*OsSPL7*

%	T	G	C	A	C-16	C-15	T	C-13	C-12	G	A	C	G	C	G	T	G	G	A	C	G	C	C	Indel	self-editing
A	0.0	0.0	0.0	/	2.1	0.0	0.0	0.0	0.0	0.0	/	0.0	0.0	0.0	0.0	0.0	0.0	0.0	/	0.0	0.0	0.0	0.0	16.7	4.2
T	/	0.0	0.0	0.0	10.4	85.4	/	8.3	2.1	0.0	0.0	0.0	0.0	0.0	0.0	/	0.0	0.0	0.0	0.0	0.0	0.0	0.0		
C	0.0	0.0	/	0.0	/	/	0.0	/	/	0.0	0.0	/	0.0	/	0.0	0.0	0.0	0.0	0.0	/	0.0	/	/		
G	0.0	/	0.0	0.0	0.0	0.0	0.0	0.0	0.0	/	0.0	0.0	/	0.0	/	0.0	/	/	0.0	0.0	/	0.0	0.0		

Supplemental Figure 13. Targeted cytosine base editing of *OsCOI2-T1* and *OsSPL7* in rice using rBE111a and rBE111b.

(A and B) Frequencies of nucleotide conversion across the target region in *OsCOI2-T*1 and *OsSPL7* in T0 transgenic rice lines. The first nucleotide upstream of the PAM is counted as position -1; the detected nucleotide changes are highlighted in red.

A rBE111a:*OsWx*

%	T	A	C	C	A	G	C	G	T	T	G	T	G	G	C	T	G_{-14}	A	G_{-16}	G	T	A	G	Indel	self-editing
A	0.0	/	0.0	0.0	/	0.0	0.0	0.0	0.0	0.0	0.0	0.0	0.0	0.0	0.0	0.0	50.0	/	3.1	0.0	0.0	/	0.0	3.1	21.9
T	/	0.0	0.0	0.0	0.0	0.0	0.0	0.0	/	/	0.0	/	0.0	0.0	0.0	/	0.0	0.0	0.0	0.0	/	0.0	0.0		
C	0.0	0.0	/	/	0.0	0.0	/	0.0	0.0	0.0	0.0	0.0	0.0	0.0	/	0.0	12.5	0.0	0.0	0.0	0.0	0.0	0.0		
G	0.0	0.0	0.0	0.0	0.0	/	0.0	/	0.0	0.0	/	0.0	/	/	0.0	0.0	/	0.0	/	/	0.0	0.0	/		

rBE111b:*OsWx*

%	T	A	C	C	A	G	C	G	T	T	G	T	G	G	C	T	G_{-14}	A	G	G	T	A	G	Indel	self-editing
A	0.0	/	0.0	0.0	/	0.0	0.0	0.0	0.0	0.0	0.0	0.0	0.0	0.0	0.0	0.0	15.6	/	0.0	0.0	0.0	/	0.0	0.0	4.7
T	/	0.0	0.0	0.0	0.0	0.0	0.0	0.0	/	/	0.0	/	0.0	0.0	0.0	/	0.0	0.0	0.0	0.0	/	0.0	0.0		
C	0.0	0.0	/	/	0.0	0.0	/	0.0	0.0	0.0	0.0	0.0	0.0	0.0	/	0.0	6.3	0.0	0.0	0.0	0.0	0.0	0.0		
G	0.0	0.0	0.0	0.0	0.0	/	0.0	/	0.0	0.0	/	0.0	/	/	0.0	0.0	/	0.0	/	/	0.0	0.0	/		

B rBE111a:*OsCOI2-T2*

%	C	T	G	G	T	G	C	A	G	G	G	T	C	G_{-11}	A	C	G_{-14}	C	G_{-16}	C	T	C	A	Indel	self-editing
A	0.0	0.0	0.0	0.0	0.0	0.0	0.0	/	0.0	0.0	0.0	0.0	0.0	33.3	/	0.0	10.4	0.0	6.3	0.0	0.0	0.0	/	6.3	6.2
T	0.0	/	0.0	0.0	/	0.0	0.0	0.0	0.0	0.0	0.0	/	0.0	0.0	0.0	0.0	0.0	0.0	0.0	0.0	/	0.0	0.0		
C	/	0.0	0.0	0.0	0.0	0.0	/	0.0	0.0	0.0	0.0	0.0	/	6.3	0.0	/	0.0	/	0.0	/	0.0	/	0.0		
G	0.0	0.0	/	/	0.0	/	0.0	0.0	/	/	/	0.0	0.0	/	0.0	0.0	/	0.0	/	0.0	0.0	0.0	0.0		

rBE111b:*OsCOI2-T2*

%	C	T	G	G	T	G	C	A	G	G	G	T	C	G_{-11}	A	C	G	C	G	C	T	C	A	Indel	self-editing
A	0.0	0.0	0.0	0.0	0.0	0.0	0.0	/	0.0	0.0	0.0	0.0	0.0	8.3	/	0.0	0.0	0.0	0.0	0.0	0.0	0.0	/	10.4	4.2
T	0.0	/	0.0	0.0	/	0.0	0.0	0.0	0.0	0.0	0.0	/	0.0	0.0	0.0	0.0	0.0	0.0	0.0	0.0	/	0.0	0.0		
C	/	0.0	0.0	0.0	0.0	0.0	/	0.0	0.0	0.0	0.0	0.0	/	0.0	0.0	/	0.0	/	0.0	/	0.0	/	0.0		
G	0.0	0.0	/	/	0.0	/	0.0	0.0	/	/	/	0.0	0.0	/	0.0	0.0	/	0.0	/	0.0	0.0	0.0	0.0		

Supplemental Figure 14. Targeted cytosine base editing of *OsWx* and *OsCOI2-T2* in rice using rBE111a and rBE111b.

(A and B) Frequencies of nucleotide conversion across the target region in *OsWx* and *OsCOI2-T2* in T0 transgenic rice lines. The first nucleotide upstream of the PAM is counted as position -1; the detected

A rBE112a:*OsCOI2-T3*

%	C	C	G	C	C	A	C	T	G	G	T	G	C	A	G_{-12}	G_{-13}	G_{-14}	T	C	G	A	C	G	Indel
A	0.0	0.0	0.0	0.0	0.0	/	0.0	0.0	0.0	0.0	0.0	0.0	0.0	/	6.3	27.1	6.3	0.0	0.0	0.0	/	0.0	0.0	4.2
T	0.0	0.0	0.0	0.0	0.0	0.0	0.0	/	0.0	0.0	/	0.0	0.0	0.0	0.0	0.0	0.0	/	0.0	0.0	0.0	0.0	0.0	
C	/	/	0.0	/	/	0.0	/	0.0	0.0	0.0	0.0	0.0	/	0.0	0.0	6.3	0.0	0.0	/	0.0	0.0	/	0.0	
G	0.0	0.0	/	0.0	0.0	0.0	0.0	0.0	/	/	0.0	/	0.0	0.0	/	/	/	0.0	0.0	/	0.0	0.0	/	

B rBE112b:*OsCOI2-T3*

%	C	C	G	C	C	A	C	T	G	G	T	G	C	A	G_{-12}	G_{-13}	G_{-14}	T	C	G	A	C	G	Indel
A	0.0	0.0	0.0	0.0	0.0	/	0.0	0.0	0.0	0.0	0.0	0.0	0.0	/	4.2	6.3	4.2	0.0	0.0	0.0	/	0.0	0.0	75.0
T	0.0	0.0	0.0	0.0	0.0	0.0	0.0	/	0.0	0.0	/	0.0	0.0	0.0	0.0	4.2	0.0	/	0.0	0.0	0.0	0.0	0.0	
C	/	/	0.0	/	/	0.0	/	0.0	0.0	0.0	0.0	0.0	/	0.0	0.0	35.4	0.0	0.0	/	0.0	0.0	/	0.0	
G	0.0	0.0	/	0.0	0.0	0.0	0.0	0.0	/	/	0.0	/	0.0	0.0	/	/	/	0.0	0.0	/	0.0	0.0	/	

C

rBE112a//gOsCOI2-T3 NGG PAM

```
  sgRNA 5'-CCACUGGUGCAGGGUCGACG-3'
5'-GTGTGCCGCCACTGGTGCAGGGTCGACGCGCTC-3'     WT
5'-GTGTGCCGCCACTGGTGCAGAGTCGACGCGCTC-3'   9/48 18.8%
5'-GTGTGCCGCCACTGGTGCAAAATCGACGCGCTC-3'   2/48  4.2%
5'-GTGTGCCGCCACTGGTGCAGCGTCGACGCGCTC-3'   3/48  6.3%
5'-GTGTGCCGCCACTGGTGCAAAGTCGACGCGCTC-3'   1/48  2.1%
5'-GTGTGCCGCCACTGGTGCAGAATCGACGCGCTC-3'   1/48  2.1%
5'-GTGTGCCGCCACTGGTGCAG--TCGACGCGCTC-3'   1/48  2.1%
5'-GTGTGCCGCCACTG--------TCGACGCGCTC-3'   1/48  2.1%
```

D

rBE112b//gOsCOI2-T3 NGG PAM

```
  sgRNA 5'-CCACUGGUGCAGGGUCGACG-3'
5'-GTGTGCCGCCACTGGTGCAGGGTCGACGCGCTC-3'     WT
5'-GTGTGCCGCCACTGGTGCAGCGTCGACGCGCTC-3'  16/48 33.3%
5'-GTGTGCCGCCACTGGTGCAGAGTCGACGCGCTC-3'   2/48  4.2%
5'-GTGTGCCGCCACTGGTGCAGTATCGACGCGCTC-3'   2/48  4.2%
5'-GTGTGCCGCCACTGGTGCAGGGgTCGACGCGCT-3'   2/48  4.2%
5'-GTGTGCCGCCACTGGTGCAACGTCGACGCGCTC-3'   1/48  2.1%
5'-GTGTGCCGCCACTGGTGCAAAGTCGACGCGCTC-3'   1/48  2.1%
5'-GTGTGCCGCCACTGGTGCAGGGTgcagggtGCG-3'   1/48  2.1%
5'-GTGTGCCGCCACcTGGTGCAGGGTCGACGCGCT-3'   1/48  2.1%
5'-GTGTGCCGCCACTGGTGCgcAGGGTCGACGCGC-3'   1/48  2.1%
5'-GTGTGCCGCCA---(-10)--GTCGACGCGCTC-3'   1/48  2.1%
5'-GTGTGCCGCCAC------AGGGTCGACGCGCTC-3'   6/48 12.5%
5'-GTGTGCCGCCACTGG------GTCGACGCGCTC-3'   1/48  2.1%
5'-GTGTGCCGCCACTGcacC----TCGACGCGCTC-3'   1/48  2.1%
5'-GTGTGCCGCCACTGGTGCAGG-TCGACGCGCTC-3'   2/48  4.2%
5'--------(-19)--------GGTCGACGCGCTC-3'   1/48  2.1%
5'-GTGTGCCGCCACTG---CAGGGTCGACGCGCTC-3'   1/48  2.1%
5'-GTGTGCCGCCA---(-11)---TCGACGCGCTC-3'   3/48  6.3%
5'-GTGTGCCGCCACTGGTGCAGGtgcaggGCGCTC-3'   1/48  2.1%
5'-GTGTGCCGCCACTGGTGC-(-6)-GACGCGCTC-3'   2/48  4.2%
5'-GTGTGCCGCCACTG---CAccGTCGACGCGCTC-3'   1/48  2.1%
5'-GTGTGCCGCCACTGGTGCtggtgcAGGGCGCTC-3'   1/48  2.1%
5'-GTGTGCCGCCACTGGT--AGGGTCGACGCGCTC-3'   1/48  2.1%
5'-GTGTGCCGCCACTGGTGCAG--TCGACGCGCTC-3'   1/48  2.1%
5'-GTGTGCCGCC----(-12)---TCGACGCGCTC-3'   1/48  2.1%
5'-GTGTGCCGCCA-----GCAGGGTCGACGCGCTC-3'   1/48  2.1%
5'-GTGTGCCGCCA----TGCAGGGTCGACGCGCTC-3'   1/48  2.1%
5'-GTGTGCCGCCACTGGTGCAGctggtgcagGCTC-3'   1/48  2.1%
5'-GTGTGCCGCCAC-(-7)--GGGTCGACGCGCTC-3'   1/48  2.1%
5'-GTGTGCCGCCACTGc---(-10)--ACGCGCTC-3'   1/48  2.1%
5'-GTGTGCCGCCACTGGTGCtgcAGGGTCGCGCTC-3'   1/48  2.1%
5'-GTGTGCCGCCACTG---CAGGGTCGACGCGCTC-3'   1/48  2.1%
5'-GTGTGCCGCCA---------(-23)---------3'   1/48  2.1%
5'-GTGTGCCGCCAC------(-15)----GCGCTC-3'   1/48  2.1%
5'-GTGTGCCGCCAC---------GTCGACGCGCTC-3'   1/48  2.1%
5'-GTGTGCCGC----------(-30)----------3'   1/48  2.1%
5'-GTGTGCCGCC--(-9)---GGGTCGACGCGCTC-3'   1/48  2.1%
5'-GTGTGCCGCCA---(-10)--GTCGACGCGCTC-3'   2/48  4.2%
5'-GTGTGCCGCCACTGGT-------CGACGCGCTC-3'   1/48  2.1%
5'-GTGTGCCGCCACTGGTGCA-------CGCGCTC-3'   1/48  2.1%
```

Supplemental Figure 15. Targeted C-to-G base editing of *OsCOI2-T3* in rice using rBE112a and rBE112b.

(A and B) Frequencies of nucleotide conversion across the target region in *OsCOI2-T3* in T0 transgenic rice lines. The first nucleotide upstream of the PAM is counted as position -1; the detected nucleotide changes are highlighted in red.

(C and D) Representative edited alleles and editing efficiency of *OsCOI2-T3* generated by each CRISPR/SpCas9n-based CGBE tool in T0 transgenic rice lines. The PAM sequences, target bases in the editing window, and detected nucleotide changes are highlighted in green, red, and blue, respectively.

A **rBE112a:*OsJAR2***

%	C	G	G	A	T	C-15	C	T	C	G	C	C	G	A	G	A	A	C	G	G	C	G	G	Indel
A	0.0	0.0	0.0	/	0.0	0.0	0.0	0.0	0.0	0.0	0.0	0.0	0.0	/	0.0	/	/	0.0	0.0	0.0	0.0	0.0	0.0	5.0
T	0.0	0.0	0.0	0.0	/	25.0	0.0	/	0.0	0.0	0.0	0.0	0.0	0.0	0.0	0.0	0.0	0.0	0.0	0.0	0.0	0.0	0.0	
C	/	0.0	0.0	0.0	0.0	/	/	0.0	/	0.0	/	/	0.0	0.0	0.0	0.0	0.0	/	0.0	0.0	/	0.0	0.0	
G	0.0	/	/	0.0	0.0	12.5	0.0	0.0	0.0	/	0.0	0.0	/	0.0	/	0.0	0.0	0.0	/	/	0.0	/	/	

B **rBE112b:*OsJAR2***

%	C	G	G	A	T	C-15	C-14	T	C-12	G	C	C	G	A	G	A	A	C	G	G	C	G	G	Indel
A	0.0	0.0	0.0	/	0.0	5.3	0.0	0.0	0.0	0.0	0.0	0.0	0.0	/	0.0	/	/	0.0	0.0	0.0	0.0	0.0	0.0	21.1
T	0.0	0.0	0.0	0.0	/	21.1	15.8	/	0.0	0.0	0.0	0.0	0.0	0.0	0.0	0.0	0.0	0.0	0.0	0.0	0.0	0.0	0.0	
C	/	0.0	0.0	0.0	0.0	/	/	0.0	/	0.0	/	/	0.0	0.0	0.0	0.0	0.0	/	0.0	0.0	/	0.0	0.0	
G	0.0	/	/	0.0	0.0	50	0.0	0.0	5.3	/	0.0	0.0	/	0.0	/	0.0	0.0	0.0	/	/	0.0	/	/	

C

rBE112a/gOsJAR2 NGG PAM

```
  sgRNA 5′-CGGAUCCUCGCCGAGAACGG-3′
5′-CGCTGCGGCGGATCCTCGCCGAGAACGGCGGCG-3′      WT
5′-CGCTGCGGCGGATTCTCGCCGAGAACGGCGGCG-3′   10/40 25.0%
5′-CGCTGCGGCGGATGCTCGCCGAGAACGGCGGCG-3′    5/40 12.5%
5′-CGCTGCGGCGGAT--(-11)----ACGGCGGCG-3′    1/40  2.5%
5′-CGCTGCGGCGGATCCTCGCCGAGAAC--(-15)-3′    1/40  2.5%
```

D

rBE112b/gOsJAR2 NGG PAM

```
  sgRNA 5′-CGGAUCCUCGCCGAGAACGG-3′
5′-CGCTGCGGCGGATCCTCGCCGAGAACGGCGGCG-3′     WT
5′-CGCTGCGGCGGATGCTCGCCGAGAACGGCGGCG-3′   17/38 44.7%
5′-CGCTGCGGCGGATTCTCGCCGAGAACGGCGGCG-3′    4/38 10.5%
5′-CGCTGCGGCGGATTTTCGCCGAGAACGGCGGCG-3′    3/38  7.9%
5′-CGCTGCGGCGGATATTCGCCGAGAACGGCGGCG-3′    2/38  5.3%
5′-CGCTGCGGCGGATGCTGGCCGAGAACGGCGGCG-3′    2/38  5.3%
5′-CGCTGCGGCGGATGTTCGCCGAGAACGGCGGCG-3′    1/38  2.7%
5′-CGCTGCGGCGGATg-TCGCCGAGAACGGCGGCG-3′    1/38  2.7%
5′-CGCTGCGGCGGAT---(-10)--AACGGCGGCG-3′    1/38  2.7%
5′-CGCTGCGGCGGATtg--GCCGAGAACGGCGGCG-3′    1/38  2.7%
5′-CGCTGCGGCGGATTGGCCGAA--AACGGCGGCG-3′    1/38  2.7%
5′-CGCTGCGGCGGA---TCGCCGAGAACGGCGGCG-3′    1/38  2.7%
5′-CGCTGCGGCGGA--(-8)--GAGAACGGCGGCG-3′    2/38  5.3%
5′-CGCTGCGGCGGATt-TCGCCGAGAACGGCGGCG-3′    1/38  2.7%
5′-CGCTGCGGCGGA---TCGCCGAGAACGGCGGCG-3′    1/38  2.7%
5′-CGCTGCGGCGGATC--CGCCGAGAACGGCGGCG-3′    1/38  2.7%
5′-CGCTGCGGCGGATC---(-10)--ACGGCGGCG-3′    1/38  2.7%
```

Supplemental Figure 16. Targeted C-to-G base editing of *OsJAR2* in rice using rBE112a and rBE112b. **(A and B)** Frequencies of nucleotide conversion across the target region in *OsJAR2* in T0 transgenic rice lines. The first nucleotide upstream of the PAM is counted as position -1; the detected nucleotide changes are highlighted in red.

(C and D) Representative edited alleles and editing efficiency of *OsJAR2* generated by each CRISPR/SpCas9n-based CGBE tool in T0 transgenic rice lines. The PAM sequences, target bases in the editing window, and detected nucleotide changes are highlighted in green, red, and blue, respectively.

A rBE112a:*OsALS1*

%	C	C	T	A	T	G	A	T	C	C	C	A	A	G	T	G_{-13}	G_{-14}	G_{-15}	G_{-16}	G_{-17}	C	G	C	Indel
A	0.0	0.0	0.0	/	0.0	0.0	/	0.0	0.0	0.0	0.0	/	0.0	0.0	0.0	2.1	2.1	62.5	79.2	2.1	0.0	0.0	0.0	43.8
T	0.0	0.0	/	0.0	/	0.0	0.0	/	0.0	0.0	0.0	0.0	0.0	0.0	/	0.0	0.0	0.0	0.0	0.0	0.0	0.0	0.0	
C	/	/	0.0	0.0	0.0	0.0	0.0	0.0	/	/	/	0.0	0.0	0.0	0.0	0.0	0.0	4.2	0.0	0.0	/	0.0	/	
G	0.0	0.0	0.0	0.0	0.0	/	0.0	0.0	0.0	0.0	0.0	0.0	0.0	/	0.0	/	/	/	/	/	0.0	/	0.0	

B rBE112b:*OsALS1*

%	C	C	T	A	T	G	A	T	C	C	C	A	A	G	T	G	G_{-14}	G_{-15}	G_{-16}	G_{-17}	C	G	C	Indel
A	0.0	0.0	0.0	/	0.0	0.0	/	0.0	0.0	0.0	0.0	/	0.0	0.0	0.0	0.0	2.1	6.3	4.2	2.1	0.0	0.0	0.0	31.3
T	0.0	0.0	/	0.0	/	0.0	0.0	/	0.0	0.0	0.0	0.0	0.0	0.0	/	0.0	0.0	0.0	2.1	0.0	0.0	0.0	0.0	
C	/	/	0.0	0.0	0.0	0.0	0.0	0.0	/	/	/	0.0	0.0	0.0	0.0	0.0	6.3	2.1	12.5	0.0	/	0.0	/	
G	0.0	0.0	0.0	0.0	0.0	/	0.0	0.0	0.0	0.0	0.0	0.0	0.0	/	0.0	/	/	/	/	/	0.0	/	0.0	

C

rBE112a/gOsALS1 NGG PAM

```
  sgRNA 5′-AUGAUCCCAAGUGGGGGCGC-3′
5′-TGCTGCCTATGATCCCAAGTGGGGGCGCATTCA-3′       WT
5′-TGCTGCCTATGATCCCAAGTGGAAGCGCATTCA-3′   24/48 50.0%
5′-TGCTGCCTATGATCCCAAGTGGGAGCGCATTCA-3′   17/48 35.4%
5′-TGCTGCCTATGATCCCAAGTGGAGGCGCATTCA-3′   10/48 20.8%
5′-TGCTGCCTATGATCCCAAGTGGAGACGCATTCA-3′    1/48  2.1%
5′-TGCTGCCTATGATCCCAAGTGGCAGCGCATTCA-3′    1/48  2.1%
5′-TGCTGCCTATGATCCCAAGTGGCGGCGCATTCA-3′    1/48  2.1%
5′-TGCTGCCTATGATCCCAAGTGAGAGCGCATTCA-3′    1/48  2.1%
5′-TGCTGCCTATGATCCCAAGTAGAGGCGCATTCA-3′    1/48  2.1%
5′-TGCTGCCTATGATCCCAAGTGGaG-CGCATTCA-3′    4/48  8.3%
5′-TGCTGCCTATGATCCCAAGTGGGG-CGCATTCA-3′    2/48  4.2%
5′-TGCTGCCTATGATCCCAAG----GGCGCATTCA-3′    1/48  2.1%
5′-TGCTGCCTATGATCC----TGGGaGCGCATTCA-3′    2/48  4.2%
5′-TGCTGCCTATGATCCCAAGTGcaagtgGGGGCG-3′    1/48  2.1%
5′-TGCTGCCTATGATCCCAAG-----GCGCATTCA-3′    1/48  2.1%
5′-TGCTGCCTATGATCCCAAGTGcGGaGCGCATTC-3′    1/48  2.1%
5′-TGCTGCCTATGATCC-AAGTGGGGGCGCATTCA-3′    1/48  2.1%
5′----------------(-49)--------------3′    1/48  2.1%
5′-TGCTGCCT----(-17)--------CGCATTCA-3′    1/48  2.1%
5′-TGCTGCCTATGATCCCAAGTGG---CGCATTCA-3′    1/48  2.1%
5′-TGCTGCCTATGATa--(-10)---GCGCATTCA-3′    1/48  2.1%
5′-TGCTGCCTATGATCCCAAGTGGGaccaagtggg-3′    1/48  2.1%
5′-TGCTGCCTATG--(-10)---GGaGCGCATTCA-3′    1/48  2.1%
5′-TGCTGCCTATGATCCCAAGTGGGaGGCGCATTC-3′    1/48  2.1%
5′-TGCTGCCTATGATCCCAAGTGGcaagtggGGGC-3′    1/48  2.1%
```

D

rBE112b/gOsALS1 NGG PAM

```
  sgRNA 5′-AUGAUCCCAAGUGGGGGCGC-3′
5′-TGCTGCCTATGATCCCAAGTGGGGGCGCATTCA-3′      WT
5′-TGCTGCCTATGATCCCAAGTGGGCGCGCATTCA-3′    3/48 6.3%
5′-TGCTGCCTATGATCCCAAGTGGAAGCGCATTCA-3′    2/48 4.2%
5′-TGCTGCCTATGATCCCAAGTGGGCACGCATTCA-3′    1/48 2.1%
5′-TGCTGCCTATGATCCCAAGTGCGCGCGCATTCA-3′    1/48 2.1%
5′-TGCTGCCTATGATCCCAAGTGCGAGCGCATTCA-3′    1/48 2.1%
5′-TGCTGCCTATGATCCCAAGTGGATGCGCATTCA-3′    1/48 2.1%
5′-TGCTGCCTATGATCCCAAGTGCCGGCGCATTCA-3′    1/48 2.1%
5′-TGCTGC--------(-20)-------GCATTCA-3′    1/48 2.1%
5′-TGCTG---------(-22)--------CATTCA-3′    1/48 2.1%
5′-TGCTGCCTATGATCCCAAGTGGatcccaagtgg-3′    1/48 2.1%
5′-TGCTGCC--------(-20)-------CATTCA-3′    1/48 2.1%
5′-TGCTGCCTATGATCCCAAGTGGtggGGGCGCAT-3′    1/48 2.1%
5′-TGCTGCCTATGATCCCAAGTGGGG-CGCATTCA-3′    1/48 2.1%
5′--------(-37)--------GGGCGCGCATTCA-3′    1/48 2.1%
5′--------(-31)--------GGGCGCGCATTCA-3′    1/48 2.1%
5′-TGCTGCCTATGATCCC------GGGCGCATTCA-3′    1/48 2.1%
5′-TGCTGCCTATGA--------GGGGGCGCATTCA-3′    1/48 2.1%
5′-TGCTGCCTATGATCCCAAGTG----CGCATTCA-3′    1/48 2.1%
5′-TGCTGCCTATGA---(-12)----GCGCATTCA-3′    1/48 2.1%
5′-TGCTGCCTATG--------TGGGGGCGCATTCA-3′    1/48 2.1%
5′-TGCTGCCTATGATCCCAAGTGagtgGGGCGCAT-3′    1/48 2.1%
5′-TGCTGCCTATGATCCCAAG------CGCATTCA-3′    1/48 2.1%
5′---------(-31)------------CGCATTCA-3′    1/48 2.1%
```

Supplemental Figure 17. Targeted C-to-G base editing of *OsALS1* in rice using rBE112a and rBE112b. **(A and B)** Frequencies of nucleotide conversion across the target region in *OsALS1* in T0 transgenic rice lines. The first nucleotide upstream of the PAM is counted as position -1; the detected nucleotide changes are highlighted in red.

(C and D) Representative edited alleles and editing efficiency of *OsALS1* generated by each CRISPR/SpCas9n-based CGBE tool in T0 transgenic rice lines. The PAM sequences, target bases in the editing window, and detected nucleotide changes are highlighted in green, red, and blue, respectively.

A **rBE112a:*OsAOS1***

%	C	G	G	C_{-17}	C_{-16}	G	C_{-14}	G	A	C	G	G	C	T	T	C	T	T	C	G	C	G	G	Indel
A	0.0	0.0	0.0	0.0	0.0	0.0	0.0	0.0	/	0.0	0.0	0.0	0.0	0.0	0.0	0.0	0.0	0.0	0.0	0.0	0.0	0.0	0.0	0.0
T	0.0	0.0	0.0	2.5	10.0	0.0	2.5	0.0	0.0	0.0	0.0	0.0	0.0	/	/	0.0	/	/	0.0	0.0	0.0	0.0	0.0	
C	/	0.0	0.0	/	/	0.0	/	0.0	0.0	/	0.0	0.0	/	0.0	0.0	/	0.0	0.0	/	0.0	/	0.0	0.0	
G	0.0	/	/	0.0	0.0	/	0.0	/	0.0	0.0	/	/	0.0	0.0	0.0	0.0	0.0	0.0	0.0	/	0.0	/	/	

B **rBE112b:*OsAOS1***

%	C	G	G	C_{-17}	C_{-16}	G	C_{-14}	G	A	C	G	G	C	T	T	C	T	T	C	G	C	G	G	Indel
A	0.0	0.0	0.0	0.0	0.0	0.0	0.0	0.0	/	0.0	0.0	0.0	0.0	0.0	0.0	0.0	0.0	0.0	0.0	0.0	0.0	0.0	0.0	37.5
T	0.0	0.0	0.0	2.5	10	2.5	0.0	0.0	0.0	0.0	0.0	0.0	0.0	/	/	0.0	/	/	0.0	0.0	0.0	0.0	0.0	
C	/	0.0	0.0	/	/	0.0	/	0.0	0.0	/	0.0	0.0	/	0.0	0.0	/	0.0	0.0	/	0.0	/	0.0	0.0	
G	0.0	/	/	0.0	2.5	/	7.5	/	0.0	0.0	/	/	0.0	0.0	0.0	0.0	0.0	0.0	0.0	/	0.0	/	/	

C

rBE112a//gOsAOS1 NGG PAM

```
  sgRNA 5'-CGGCCGCGACGGCUUCUUCG-3'
5'-GGCCCGGGCGGCCGCGACGGCTTCTTCGCGGCG-3'   WT
5'-GGCCCGGGCGGCTGCGACGGCTTCTTCGCGGCG-3'  2/40  5.0%
5'-GGCCCGGGCGGCTGTGACGGCTTCTTCGCGGCG-3'  1/40  2.5%
5'-GGCCCGGGCGGTTGTGACGGCTTCTTCGCGGCG-3'  1/40  2.5%
```

D

rBE112b//gOsAOS1 NGG PAM

```
  sgRNA 5'-CGGCCGCGACGGCUUCUUCG-3'
5'-GGCCCGGGCGGCCGCGACGGCTTCTTCGCGGCG-3'   WT
5'-GGCCCGGGCGGCTGCGACGGCTTCTTCGCGGCG-3'  4/40  10.0%
5'-GGCCCGGGCGGCCGGGACGGCTTCTTCGCGGCG-3'  2/40  5.0%
5'-GGCCCGGGCGGCGGGGACGGCTTCTTCGCGGCG-3'  1/40  2.5%
5'-GGCCCGGGCGGCCTCGACGGCTTCTTCGCGGCG-3'  1/40  2.5%
5'-GGCCCGGGCGGTCGCGACGGCTTCTTCGCGGCG-3'  1/40  2.5%
5'-GGCCCGGGCGGC-------(-20)--------G-3'  1/40  2.5%
5'-GGCCCGGGCGGC---GACGGCTTCTTCGCGGCG-3'  2/40  5.0%
5'-GGCCCGGGCGG--------(-22)----------3'  1/40  2.5%
5'-GGCCCGGGCGGCCGC------TTCTTCGCGGCG-3'  2/40  5.0%
5'-GGCCCGGGCGGCCGCGA--------TCGCGGCG-3'  1/40  2.5%
5'-GGCCCGGGCGGCCGCGACGGCTTCTTggcttct-3'  1/40  2.5%
5'-GGCCCGGGCGGCCGCGACGGacggCTTCTTCCG-3'  1/40  2.5%
5'-GGCCCGGGCGGCCG---(-10)--TTCGCGGCG-3'  1/40  2.5%
5'-GGCCCGGGCG-----GACGGCTTCTTCGCGGCG-3'  1/40  2.5%
5'-GGCCCGGGCG------ACGGCTTCTTCGCGGCG-3'  2/40  5.0%
5'-GGCCCGGGCGGCCGCG-----(-14)----GCG-3'  1/40  2.5%
5'-GGCCCGGGCGG-----ACGGCTTCTTCGCGGCG-3'  1/40  2.5%
5'-GGCCCGGGCGGCCGCGACGGCgacggcTTCTCG-3'  1/40  2.5%
5'-GGCCCGGGCGGCCGCG----(-12)---CGGCG-3'  1/40  2.5%
```

Supplemental Figure18. Targeted C-to-G base editing of *OsAOS1* in rice using rBE112a and rBE112b. (A and B) Frequencies of nucleotide conversion across the target region in *OsAOS1* in T0 transgenic rice lines. The first nucleotide upstream of the PAM is counted as position -1; the detected nucleotide changes are highlighted in red.

(C and D) Representative edited alleles and editing efficiency of *OsAOS1* generated by each CRISPR/SpCas9n-based CGBE tool in T0 transgenic rice lines. The PAM sequences, target bases in the editing window, and detected nucleotide changes are highlighted in green, red, and blue, respectively.

A **rBE114a:*OsGS1-T2***

%	C	C	T	T	G	G	C	T	G	G	C	C	T_{-10}	G	T	T_{-13}	G_{-14}	G	T_{-16}	G_{-17}	G	C	T	Indel
A	0.0	0.0	0.0	0.0	0.0	0.0	0.0	0.0	0.0	0.0	0.0	0.0	0.0	0.0	0.0	0.0	89.6	0.0	0.0	12.5	0.0	0.0	0.0	10.4
T	0.0	0.0	/	/	0.0	0.0	0.0	/	0.0	0.0	0.0	0.0	/	0.0	/	/	0.0	0.0	/	0.0	0.0	0.0	/	
C	/	/	0.0	0.0	0.0	0.0	/	0.0	0.0	0.0	/	/	6.3	0.0	0.0	20.8	10.4	0.0	95.8	0.0	0.0	/	0.0	
G	0.0	0.0	0.0	0.0	/	/	0.0	0.0	/	/	0.0	0.0	0.0	/	0.0	0.0	/	/	0.0	/	/	0.0	0.0	

B **rBE114a:*OsWRKY45-T2***

%	G	A	T	C_{-17}	A_{-16}	C_{-15}	C_{-14}	G	A	A	G	A	A	T	C	A	T	G	G	A	T	G	G	Indel
A	0.0	/	0.0	0.0	/	0.0	0.0	0.0	/	/	0.0	/	/	0.0	0.0	/	0.0	0.0	0.0	/	0.0	0.0	0.0	45.8
T	0.0	0.0	/	10.4	0.0	29.2	62.5	0.0	0.0	0.0	0.0	0.0	0.0	/	0.0	0.0	/	0.0	0.0	0.0	/	0.0	0.0	
C	0.0	0.0	0.0	/	0.0	/	/	0.0	0.0	0.0	0.0	0.0	0.0	0.0	/	0.0	0.0	0.0	0.0	0.0	0.0	0.0	0.0	
G	/	0.0	0.0	2.1	85.4	2.1	4.2	/	0.0	0.0	/	0.0	0.0	0.0	0.0	0.0	0.0	/	/	0.0	0.0	/	/	

C

rBE114a/gOsGS1-T2 NGG PAM

```
  sgRNA 5′-UGGCUGGCCUGUUGGUGGCU-3′
5′-TGGCCCCTTGGCTGGCCTGTTGGTGGCTTCCCT-3′      WT
5′-TGGCCCCTTGGCTGGCCTGTTAGCGGCTTCCCT-3′  42/48 87.5%
5′-TGGCCCCTTGGCTGGCCTGTTGGCGGCTTCCCT-3′  12/48 25.0%
5′-TGGCCCCTTGGCTGGCCTGTCGGCGGCTTCCCT-3′   6/48 12.5%
5′-TGGCCCCTTGGCTGGCCTGTTGGCAGCTTCCCT-3′   5/48 10.4%
5′-TGGCCCCTTGGCTGGCCTGTTCGCGGCTTCCCT-3′   4/48  8.3%
5′-TGGCCCCTTGGCTGGCCTGTCAGTGGCTTCCCT-3′   3/48  6.3%
5′-TGGCCCCTTGGCTGGCCCGTTAGTGGCTTCCCT-3′   2/48  4.2%
5′-TGGCCCCTTGGCTGGCCTGTTAGTGGCTTCCCT-3′   2/48  4.2%
5′-TGGCCCCTTGGCTGGCCCGTTGGTAGCTTCCCT-3′   1/48  2.1%
5′-TGGCCCCTTGGCTGGCCTGTCAGAAGCTTCCCT-3′   1/48  2.1%
5′-TGGCCCCTTGGCTGGCCTGTTACCGGCTTCCCTC-3′  1/48  2.1%
5′-TGGCCCCTTGGCTGGCCTGTTGGTCGCTTCCCT-3′   1/48  2.1%
5′-TGGCCCCTTGGCTGGCCTGCTGGCGGCTTCCCT-3′   1/48  2.1%
5′-TGGCCCCTTGGCTGGCCTGTTTGGCGGCTTCCC-3′   1/48  2.1%
5′-TGGCCCCTTGGCTGGCCTGTTCGTGGCTTCCCT-3′   1/48  2.1%
5′-TGGCCCCTTGGCTGGCCCGTTGGTAGCTTCCCT-3′   1/48  2.1%
5′-TGGCCCCTTGGCTG---------TGGCTTCCCT-3′   1/48  2.1%
5′-TGGCCCCTTGGCTGGCCTGTTG-TGGCTTCCCT-3′   1/48  2.1%
5′-TGGCCCCTTGGCTGGCCTGTTGc-GGCTTCCCT-3′   1/48  2.1%
5′-TGGCCCCTTGGCTGGC--------GGCTTCCCT-3′   1/48  2.1%
5′-TGGCCCCTTGGCTGGCCTGTTGGcaGCTTCCCT-3′   1/48  2.1%
```

D

rBE114a//gOsWRKY45-T2 NGG PAM

```
  sgRNA 5′-GAUCACCGAAGAAUCAUGGA-3′
5′-GAGCTTTTGATCACCGAAGAATCATGGATGGAC-3′      WT
5′-GAGCTTTTGATCGCTGAAGAATCATGGATGGAC-3′  28/48 58.3%
5′-GAGCTTTTGATCGTCGAAGAATCATGGATGGAC-3′   7/48 14.6%
5′-GAGCTTTTGATCGTTGAAGAATCATGGATGGAC-3′   5/48 10.4%
5′-GAGCTTTTGATCGCCGAAGAATCATGGATGGAC-3′   4/48  8.3%
5′-GAGCTTTTGATTGCCGAAGAATCATGGATGGAC-3′   4/48  8.3%
5′-GAGCTTTTGATCATTGAAGAATCATGGATGGAC-3′   2/48  4.2%
5′-GAGCTTTTGATCGCGGAAGAATCATGGATGGAC-3′   2/48  4.2%
5′-GAGCTTTTGATCAGCGAAGAATCATGGATGGAC-3′   1/48  2.1%
5′-GAGCTTTTGATGGCCGAAGAATCATGGATGGAC-3′   1/48  2.1%
5′-GAGCTTTTGATTGTTGAAGAATCATGGATGGAC-3′   1/48  2.1%
5′-GAGCTTTTGATCgC-GAAGAATCATGGATGGAC-3′   2/48  4.2%
5′-GAGCTTTTGATC---GAAGAATCATGGATGGAC-3′   1/48  2.1%
5′-GAGCTTTTGATt---GAAGAATCATGGATGGAC-3′   1/48  2.1%
5′-GAGCTTTTGATCgCC-AAGAATCATGGATGGAC-3′   1/48  2.1%
5′-GAGCTTTTGAT----GAAGAATCATGGATGGAC-3′   2/48  4.2%
5′-GAGCTTTTGATCAC---------ATGGATGGAC-3′   1/48  2.1%
5′-GAGCTTTTGATCgCC---------TGGATGGAC-3′   1/48  2.1%
5′-GAGCTTTTGATgCC---------ATGGATGGAC-3′   1/48  2.1%
5′-GAGCTTTTGATCACCG--(-10)---GATGGAC-3′   2/48  4.2%
5′-GAGCTTTTGATCgC---(-10)--TGGATGGAC-3′   1/48  2.1%
5′-GAGCTTTTGATCgCCG--(-10)---GATGGAC-3′   1/48  2.1%
5′-GAGCTTTTGATCAC---(-11)---GGATGGAC-3′   3/48  6.3%
5′-GAGCTTTTGATCgC---(-11)---GGATGGAC-3′   1/48  2.1%
5′-GAGCTTTTGATCgCCGA--(-11)----TGGAC-3′   1/48  2.1%
5′-GAGCTTTTGATtgC---(-11)---GGATGGAC-3′   1/48  2.1%
5′-GAGCTTTTGATCg----(-12)---GGATGGAC-3′   1/48  2.1%
5′-GAGCTTTTGAT---(-14)------GGATGGAC-3′   1/48  2.1%
5′-GAGCTTTTGATCgCgGt------(-15)----G-3′   1/48  2.1%
5′-GAGCTTTTGATC----(-13)----GGATGGAC-3′   1/48  2.1%
5′-GAGCTTTTt-------(-22)---------GAC-3′   1/48  2.1%
5′-GAGCTTTTGATCgCCGAA------(-25)-----3′   1/48  2.1%
```

Supplemental Figure 19. Dual cytosine and adenine base editing of *OsGS1-T2* and *OsWRKY45-T2* in rice using rBE114a.

(A and B) Frequencies of nucleotide conversion across the target region in *OsGS1-T2* and *OsWRKY45-T2* in T0 transgenic rice lines. The first nucleotide upstream of the PAM is counted as position -1; the detected nucleotide changes are highlighted in red.

(C and D) Representative edited alleles and editing efficiency of *OsGS1-T2* and *OsWRKY45-T2* generated by CRISPR/SpCas9n-based dual cytosine and adenine base editing tool in T0 transgenic rice lines. The PAM sequences, target bases in the editing window, and detected nucleotide changes are highlighted in green, red, and blue, respectively.

A rBE114a:*OsTubA2*

%	C	C	G	A	G	G	A	T	C	C	A	C	T	T	C	A	T_{-14}	G_{-15}	C	T	T	T	C	Indel
A	0.0	0.0	0.0	/	0.0	0.0	/	0.0	0.0	0.0	/	0.0	0.0	0.0	0.0	/	0.0	81.3	0.0	0.0	0.0	0.0	0.0	14.6
T	0.0	0.0	0.0	0.0	0.0	0.0	0.0	/	0.0	0.0	0.0	0.0	/	/	0.0	0.0	/	0.0	0.0	/	/	/	0.0	
C	/	/	0.0	0.0	0.0	0.0	0.0	0.0	/	/	0.0	/	0.0	0.0	/	0.0	83.3	0.0	/	0.0	0.0	0.0	/	
G	0.0	0.0	/	0.0	/	/	0.0	0.0	0.0	0.0	0.0	0.0	0.0	0.0	0.0	0.0	0.0	/	0.0	0.0	0.0	0.0	0.0	

B rBE114a:*OsCOI2-T3*

%	C	C	G	C	C	A	C	T	G	G	T	G	C	A	G_{-12}	G_{-13}	G_{-14}	T_{-15}	C	G	A	C	G	Indel
A	0.0	0.0	0.0	0.0	0.0	/	0.0	0.0	0.0	0.0	0.0	0.0	0.0	/	12.5	89.6	6.3	0.0	0.0	0.0	/	0.0	0.0	25.0
T	0.0	0.0	0.0	0.0	0.0	0.0	0.0	/	0.0	0.0	/	0.0	0.0	0.0	0.0	0.0	0.0	/	0.0	0.0	0.0	0.0	0.0	
C	/	/	0.0	/	/	0.0	/	0.0	0.0	0.0	0.0	0.0	/	0.0	0.0	14.6	0.0	6.3	/	0.0	0.0	/	0.0	
G	0.0	0.0	/	0.0	0.0	0.0	0.0	0.0	/	/	0.0	/	0.0	0.0	/	/	/	0.0	0.0	/	0.0	0.0	/	

C

rBE114a/gOsTubA2 NGG PAM

```
  sgRNA 5'-AGGAUCCACUUCAUGCUUUC-3'
5'-CCTACCCGAGGATCCACTTCATGCTTTCCTCCT-3'      WT
5'-CCTACCCGAGGATCCACTTCACACTTTCCTCCT-3'   39/48  81.3%
5'-CCTACCCGAGGATCCACTTCACGCTTTCCTCCT-3'   21/48  43.8%
5'-CCTACCCGAGGATCCACTT-------TCCTCCT-3'    1/48   2.1%
5'-CCTACCCGAGGATCC---------TTTCCTCCT-3'    1/48   2.1%
5'-CCTAC--------------(-30)----------3'    1/48   2.1%
5'-CCTACCCGAGGA---(-10)--GCTTTCCTCCT-3'    1/48   2.1%
5'-CCTACCCGAGGAT-----TCATGCTTTCCTCCT-3'    1/48   2.1%
5'-CCTACCCGAGGATCCACTTCATcacttcatGCT-3'    1/48   2.1%
5'-CCTACCCGAGGATCCACTTCAT-CTTTCCTCCT-3'    1/48   2.1%
```

D

rBE114a/gOsCOI2-T3 NGG PAM

```
  sgRNA 5'-CCACUGGUGCAGGGUCGACG-3'
5'-GTGTGCCGCCACTGGTGCAGGGTCGACGCGCTC-3'      WT
5'-GTGTGCCGCCACTGGTGCAGAGTCGACGCGCTC-3'   40/48  83.3%
5'-GTGTGCCGCCACTGGTGCAGCGTCGACGCGCTC-3'    7/48  14.6%
5'-GTGTGCCGCCACTGGTGCAAAGTCGACGCGCTC-3'    3/48   6.3%
5'-GTGTGCCGCCACTGGTGCAGAGCCGACGCGCTC-3'    3/48   6.3%
5'-GTGTGCCGCCACTGGTGCAGAATCGACGCGCTC-3'    2/48   4.2%
5'-GTGTGCCGCCACTGGTGCAAAATCGACGCGCTC-3'    1/48   2.1%
5'-GTGTGCCGCCACTG---CAGGGTCGACGCGCTC-3'    1/48   2.1%
5'-GTGTGCCGCCAC------AGGGTCGACGCGCTC-3'    1/48   2.1%
5'-------------(-33)-----------------3'    1/48   2.1%
5'-GTGTGCCGCCACTGcacctgGTGCAGGGTCGAC-3'    1/48   2.1%
5'-GTGTGCCGCCAC----(-15)------GCGCTC-3'    1/48   2.1%
5'-GTGTGCCGCCACTG--------TCGACGCGCTC-3'    1/48   2.1%
5'-GTGTGCCGCC---------GGGTCGACGCGCTC-3'    1/48   2.1%
5'-GTGTGCCGCCACTGGTGCtgcAGGGTCGACGCG-3'    1/48   2.1%
5'-GTGTGCCGCCACTGGTGCAGagGGTCGACGCGC-3'    1/48   2.1%
5'-GTGTGCCGCCACTGGTGCAaggtgcaGGGTCGA-3'    1/48   2.1%
5'-GTGTGCCG-------TGCAGGGTCGACGCGCTC-3'    1/48   2.1%
5'---------(-36)---------TCGACGCGCTC-3'    1/48   2.1%
5'-GTGTGCCGCCACTGGTGCAGaGcTCGACGCGCT-3'    1/48   2.1%
```

Supplemental Figure 20. Dual cytosine and adenine base editing of *OsTubA2* and *OsCOI2-T3* in rice using rBE114a.

(A and B) Frequencies of nucleotide conversion across the target region in *OsTubA2* and *OsCOI2-T3* in T0 transgenic rice lines. The first nucleotide upstream of the PAM is counted as position -1; the detected nucleotide changes are highlighted in red.

(C and D) Representative edited alleles and editing efficiency of *OsTubA2* and *OsCOI2-T3*generated by CRISPR/SpCas9n-based dual cytosine and adenine base editing tool in T0 transgenic rice lines. The PAM sequences, target bases in the editing window, and detected nucleotide changes are highlighted in green, red, and blue, respectively.

A rBE114a:*OsACC1-T1*

%	C	C	C	A	G	A	C	C	G	C	A	T	T	G_{-11}	A	G	T_{-14}	G_{-15}	C	T_{-17}	A	T	G	Indel
A	0.0	0.0	0.0	/	0.0	/	0.0	0.0	0.0	0.0	/	0.0	0.0	2.1	/	0.0	0.0	18.8	0.0	0.0	/	0.0	0.0	0.0
T	0.0	0.0	0.0	0.0	0.0	0.0	0.0	0.0	0.0	0.0	0.0	/	/	0.0	0.0	0.0	/	2.1	0.0	/	0.0	/	0.0	
C	/	/	/	0.0	0.0	0.0	/	/	0.0	/	0.0	0.0	0.0	0.0	0.0	0.0	81.3	0.0	/	50.0	0.0	0.0	0.0	
G	0.0	0.0	0.0	0.0	/	0.0	0.0	0.0	/	0.0	0.0	0.0	0.0	/	0.0	/	0.0	/	0.0	0.0	0.0	0.0	/	

B rBE114a:*OsACC1-T2*

%	C	C	T	C	G	T	G	C	T	G	G	A	C	A	A	G	T_{-14}	G_{-15}	T_{-16}	G_{-17}	G	T	T	Indel
A	0.0	0.0	0.0	0.0	0.0	0.0	0.0	0.0	0.0	0.0	0.0	/	0.0	/	/	0.0	0.0	2.1	0.0	20.8	0.0	0.0	0.0	0.0
T	0.0	0.0	/	0.0	0.0	/	0.0	0.0	/	0.0	0.0	0.0	0.0	0.0	0.0	0.0	/	0.0	/	0.0	0.0	/	/	
C	/	/	0.0	/	0.0	0.0	0.0	/	0.0	0.0	0.0	0.0	/	0.0	0.0	0.0	27.1	0.0	27.1	2.1	0.0	0.0	0.0	
G	0.0	0.0	0.0	0.0	/	0.0	/	0.0	0.0	/	/	0.0	0.0	0.0	0.0	/	0.0	/	0.0	/	/	0.0	0.0	

C

rBE114a/gOsACC1-T1 NGG PAM

```
  sgRNA 5′-AGACCGCAUUGAGUGCTAUG-3′
5′-ATAAACCCAGACCGCATTGAGTGCTATGCTGAG-3′      WT
5′-ATAAACCCAGACCGCATTGAGCGCTATGCTGAG-3′  38/48 79.2%
5′-ATAAACCCAGACCGCATTGAGTGCCATGCTGAG-3′  22/48 45.8%
5′-ATAAACCCAGACCGCATTGAGCACTATGCTGAG-3′  12/48 25.0%
5′-ATAAACCCAGACCGCATTGAGCGCCATGCTGAG-3′   2/48  4.2%
5′-ATAAACCCAGACCGCATTGAGTACTATGCTGAG-3′   2/48  4.2%
5′-ATAAACCCAGACCGCATTAAGTGCTATGCTGAG-3′   1/48  2.1%
5′-ATAAACCCAGACCGCATTGAGTTCTATGCTGAG-3′   1/48  2.2%
```

D

rBE114a/gOsACC1-T2 NGG PAM

```
  sgRNA 5′-CGUGCUGGACAAGUGUGGUU-3′
5′-CTGTTCCTCGTGCTGGACAAGTGTGGTTTCCAG-3′      WT
5′-CTGTTCCTCGTGCTGGACAAGTGCAGTTTCCAG-3′  10/48 20.8%
5′-CTGTTCCTCGTGCTGGACAAGCGCGGTTTCCAG-3′  10/48 20.8%
5′-CTGTTCCTCGTGCTGGACAAGCGTGGTTTCCAG-3′   3/48  6.3%
5′-CTGTTCCTCGTGCTGGACAAGTGCGGTTTCCAG-3′   2/48  4.2%
5′-CTGTTCCTCGTGCTGGACAAGTACGGTTTCCAG-3′   1/48  2.1%
5′-CTGTTCCTCGTGCTGGACAAGTGTCGTTTCCAG-3′   1/48  2.1%
```

Supplemental Figure 21. Dual cytosine and adenine base editing of *OsACC1-T1* and *OsACC1-T2* in rice using rBE114a.

(A and B) Frequencies of nucleotide conversion across the target region in *OsACC1-T1* and *OsACC1-T2* in T0 transgenic rice lines. The first nucleotide upstream of the PAM is counted as position -1; the detected nucleotide changes are highlighted in red.

(C and D) Representative edited alleles and editing efficiency of *OsACC1-T1* and *OsACC1-T2* generated by CRISPR/SpCas9n-based dual cytosine and adenine base editing tool in T0 transgenic rice lines. The PAM sequences, target bases in the editing window, and detected nucleotide changes are highlighted in green, red, and blue, respectively.

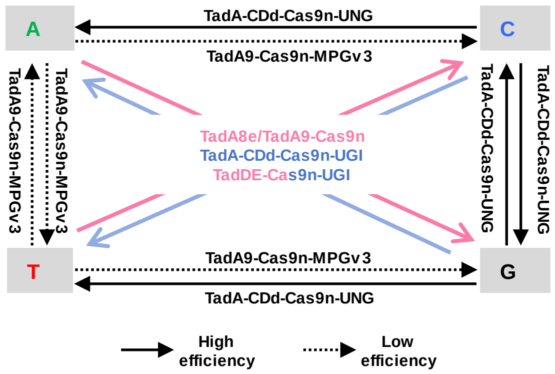

Supplemental Figure 22. Diagram showing 12 types of site-specific point mutations that can be achieved with available base editors in rice.

Supplemental Table 1. The nucleotide sequences of the rice codon-optimized *TadA-CDd*, *TadA-E27R/N46L*, *TadA-N46P*, *TadA-dual* and *OsUNG* gene fragments.

TadA-CDd	TCCGAAGTGGAGTTTAGCCACGAATATTGGATGAGACACGCTCTGACACTTGCCAAGCGCGCAAGGGACGAAAGGAAGGCCCC<br>TGTGGGCGCTGTTTTGGTGCTGAATAATAGGGTGATAGGCGAAGGATGGAATAGAGCTATAGGCCTCCATGATCCAACCGCAC<br>ATGCTGAGATAATTGCCCTTAGACAAGGAGGGCTCGTGATGCAAAATTACAGACTGATCGACGCTACTCTCTACGTTACGTTC<br>GAGCCATGCGTCATGTGCGCTGGTGCGATGATTAATTCTCGGATCGGTCGGGTTGTCTTCGGGGTGCGGAATTCCAAGCGGGG<br>CGCAGCCGGGAGTCTTATGAATGTGCTGAATTACCCTGGTATGAACCATAGGGTCGAAATTACGGAAGGAATTCTCGCCGATG<br>AATGTGCTGCCCTTTTGTGCGACTTCTACCGGATGCCGCGCCAAGTGTTTAACGCACAAAAAAAAGCACAGTCATCTATAAAT
TadA-E27R/N46L	TCAGAGGTTGAGTTCTCTCACGAGTATTGGATGAGGCACGCTCTCACTCTCGCTAAGAGGGCTAGAGATGAACGCAGGGTGCC<br>AGTTGGTGCCGTGCTTGTCTTGAACAACAGGGTGATTGGCGAGGGTTGGCTCAGGGCAATCGGATTGCATGACCCTACAGCTC<br>ATGCCGAGATTATGGCCTTGAGGCAGGGAGGGCTTGTGATGCAGAACTATAGGCTCATCGACGCAACCCTGTATGTCACCTTT<br>GAACCGTGTGTCATGTGTGCCGGTGCCATGATTCACTCTAGAATAGGGAGAGTTGTTTTCGGAGTCAGAAACTCGAAGCGCGG<br>TGCCGCAGGTAGCCTCATGAACGTGTTGAATTACCCGGGTATGAACCATCGGGTTGAAATTACCGAAGGAATACTTGCAGATG<br>AGTGCGCGGCACTCCTTTGCGATTTCTACAGGATGCCGAGGCAAGTCTTCAACGCTCAGAAGAAGGCCCAGAGTTCTATTAAT
TadA-N46P	AGCGAAGTTGAGTTCAGTCACGAGTATTGGATGCGCCACGCTCTCACCCTTGCGAAAAGGGCCAGGGACGAACGCGAAGTTCC<br>CGTTGGGGCAGTCCTCGTGCTTAACAATAGGGTGATAGGAGAGGGTTGGCCTCGCGCCATCGGACTGCATGATCCCACAGCAC<br>ATGCGGAGATCATGGCCCTGCGGCAGGGAGGATTGGTGATGCAAAATTACCGCTTGATAGATGCTACTCTCTATGTCACGTTC<br>GAGCCCTGTGTCATGTGTGCAGGCGCCATGATTCATTCTAGAATTGGTAGAGTGGTGTTCGGTGTTCGGAATTCCAAAAGAGG<br>CGCGGCGGGGTCCCTTATGAACGTCCTTAACTACCCAGGCATGAACCATCGGGTCGAGATCACCGAGGGCATACTGGCGGACG<br>AATGCGCGGCGCTCCTTTGTGATTTTTATCGGATGCCTCGGCAGGTGTTTAACGCTCAAAAGAAGGCTCAATCAAGCATAAAC
TadA-dual	AGTGAAGTGGAATTCTCGCATGAGTATTGGATGAGACACGCACTTACGTTGGCGAAACGCGCTCGCGACGAGGGAGAGGCCCC<br>TGTCGGAGCGGTTCTCGTTTTGAATAATCGCGTGATCGGAGAAGGTTGGAACAGAAGGATAGGTCTGCACGACCCAACGGCGC<br>ACGCTGAAATAATGGCACTCCGGCAGGGGGGTCTTGTCATGCAAAACTCACGCTTGATTGATGCGACTCTCTATGTTACCTTT<br>GAGCCATGCGTCATGTGTGCGGGGGCTATGATCAACTCACGCATTGGTCGGGTGGTTTTCGGGGTGCGGAACTCGAAGCGGGG<br>GGCAGCTGGTTCCCTTATGAATGTTCTGAACTACCCCGGAATGAACCACAGAGTGGAGATAACTGAAGGAATACTCGCCGACG

	AATGTGCTGCACTGTTGTGTGACTTCTACAGGATGCCGCGGCAAGTCTTCAATGCACAAAAAAAAGCGCAATCTTCCATAAAT
OsUNG	GCGCCGCCTCTCCCTCCCACCGCCCCCAAAACCATCGCCGACTACCTCATCCGCCCCTCCAAGCGCCTCCGCCCCACCTCTCC<br>CGCTCCCGCCGCCGCTGCGTCGGCCCCCCTCTCCTCCTCCAGCCTCTCGCCGGAGCAGCGCCGCCGCGCCGACACCAACCTCG<br>CGCTCGCCCGGGCGCGCCGCCACCTCCGCCTCGCCGAGTCCAAAGCGTCGGGCGGCACCGCGAAGCTGGAGGAGCTGCTCGTC<br>GAGGAGACATGGCTGGAGGCGCTTCCCGGGGAGCTGCACAAACCCTACGCGCTCGAACTCTGCCGCTTCGTCGCCCACGAGAG<br>GTTGCATAGCCCGGTGCCCGTCTACCCGCCGCCGCATCTAGTGTTCCACGCGCTTCACGCCACCCCGTTCGACCGTGTTAAGG<br>CCGTCATCATCGGGCAGGACCCATACCACGGACCTGGTCAGGCGATGGGGTTGTCTTTCTCAGTACCAGAGGGGATCAAAATT<br>CCTTCTAGCTTAGCAAACATATTTAAAGAGCTGCAAAAAGATCTAGGTTGCACCGTGCCTTCACATGGAAACTTGGAAAGATG<br>GGCTGTGCAGGGTGTTCTTATGCTCAACACTGTATTAACTGTGAGAGAACATCAAGCCAATTCACATGCCAAGAAAGGATGGG<br>AGCAATTTACTGATGCTGTCATTAAGACAATATCACTGAAGAAATCTGGAATAGTCTTTATTCTCTGGGGAAACTCAGCTCAA<br>GCAAAGACAAGATTGATTGATGAAACAAAACACCACATTTTGAAATCCGCTCATCCATCAGGGCTGTCTGCAAGCAGAGGTTT<br>CTTTGGATGCAGGCACTTTTCTAAAACGAATCAGATCTTGGAGAGGCTGGGACTATCTGCCATTGATTGGCAACTC

Note: The nucleotides causing amino acid changes from *TadA8e* are indicated in grey shadow.

Supplemental Table 2. Genome editing tools generated and used in this study.

Vector name	Editor name	Gene architecture	CRISPR system	PAM preference
pUbi:rBE110a	rBE110a	TadA-CDd-SpCas9n-UGI	SpCas9	NGG
pUbi:rBE110b	rBE110b	TadA-E27R/N46L-SpCas9n-UGI		
pUbi:rBE111a	rBE111a	TadA-CDd-SpRYn-UGI	SpRY	NRN
pUbi:rBE111b	rBE111b	TadA-E27R/N46L-SpRYn-UGI		
pUbi:rBE112a	rBE112a	TadA-N46P-SpCas9n	SpCas9	NGG
pUbi:rBE112b	rBE112b	TadA-CDd-SpCas9n-UNG		
pUbi:rBE114a	rBE114a	TadA-dual-SpCas9n-UGI		

Supplemental Table 3. Rice genes used for targeted base editing in this study.

Gene name	Gene identifier	Function description	Base editing vector used
OsCERK1	*Os08g0538300*	Chitin elicitor receptor kinase 1	pUbi:rBE110a; pUbi:rBE110b
OsJAR2	*Os01g0221100*	JA-amino acid synthetase	pUbi:rBE110a; pUbi:rBE110b; pUbi:rBE112a; pUbi:rBE112b
OsBRI1	*Os01g0718300*	Receptor serine/threonine kinase	pUbi:rBE110a; pUbi:rBE110b
OsBZR1	*Os07g0580500*	Transcription factor	pUbi:rBE110a; pUbi:rBE110b
OsFLS2	*Os04g0618700*	flg22 receptor	pUbi:rBE110a; pUbi:rBE110b
OsWRKY45	*Os05g0322900*	Transcriptional factor	pUbi:rBE110a; pUbi:rBE110b; pUbi:rBE114a
OsGS1	*Os02g0735200*	Glutamine synthetase 1	pUbi:rBE110a; pUbi:rBE110b; pUbi:rBE114a
OsGS2	*Os04g0659100*	Glutamine synthetase 2	pUbi:rBE110a; pUbi:rBE110b; pUbi:rBE114a
OsJAR1	*Os05g0586200*	JA-amino acid synthetase	pUbi:rBE110a; pUbi:rBE110b
OsAOS1	*Os03g0767000*	Allene oxide synthase	pUbi:rBE110a; pUbi:rBE110b; pUbi:rBE112a;pUbi:rBE112b
OsCOI2	*Os03g0265500*	Coronatine insensitive 2	pUbi:rBE111a;pUbi:rBE111b;pUbi:rBE112a;pUbi:rBE112b;pUbi:rBE114a

OsSPL7	*Os04g0551500*	Squamosa promoter-binding-like protein 7	pUbi:rBE111a;pUbi:rBE111b
OsWx	*Os06g0133000*	Granule-bound starch synthase	pUbi:rBE111a;pUbi:rBE111b
OsALS1	*Os02g0510200*	Acetolactate synthase 1	pUbi:rBE112a;pUbi:rBE112b
OsTubA2	*Os11g0247300*	Tubulin alpha-2	pUbi:rBE114a
OsACC1	*Os05g0295300*	acetyl-coenzyme A carboxylase 1	pUbi:rBE114a

Supplemental Table 4. List of oligonucleotides in this study.

Primer name	Primer sequence (5' - 3')	Used for
bpNLS-F1	GCTTGGATCCATGAAACGCACCGCCGAT	Fusing *TadA8e-CDd*, *TadA8e-E27R/N46L*, *TadA8e-N46P* or *TadA8e-dual* to the 5' end of *Cas9n*, respectively.
CBE1-815-R1	CCGATTGAATACTTTTTATCAGATCCCCCACTACTCCC	
bpNLS-F2	GCTTggatccATGAAAAGGACTGCGGACG	
CBE2-815-R1	CCGATTGAATACTTTTTATCAGAACCGCCGGAACTCC C	
8eN46L-2XTEN-R1	ACTACCCCCCGAGGAACC	
SpCas9(D10A)-F1	GATAAAAAGTATTCAATCGGACTTGC	
BE1-bpNLS-R1	AACTTTCCGCTTCTTTTTAGGAC	
TadAdual-F1	AGTGAAGTGGAATTCTCGCA	
pUC57-bpNLS-R1	GCGGTGCGTTTCATGGATCCAAGCTTATCTAGATGC	Generating *SpCas9n*.
OsCas9-Fg1-F4	GATAAAAAGTATTCAATCGGACTTGCTATTGGGACAA ACTCTGTG	
pUC57-bpNLS-R2	GCAGTCCTTTTCATggatccAAGCTTATCTAGATGC	
rBE66-Bcu1-R1	CTCACTAGTTCAGACCTTTCTCTTC	Generating *SpRYn*.
SpCas9(D10A)-F1	GATAAAAAGTATTCAATCGGACTTGC	
Cas9-CDd-F1	CTAGTTGGTCATAGCTGTTTCC	Generating pUbi:rBE112b.
Cas9-CDd-R1	TGAATACTTTTTATCAGATCCCCCACTACTCCCTC	

Cas9-F	GATAAAAAGTATTCAATCGG	
Cas9-R	GTCACCGCCCAACTGCGAAAG	
Cas9-UNG-F1	CAGTTGGGCGGTGACGCGCCGCCTCTCCCTCCCAC	
Cas9-UNG-R1	AAACAGCTATGACCAACTAGTTCAAACTTTCCTCTTC	
gOsCERK1-F4	tgttgTGTCCCGATGTATGTATAC	Targeting the *OsCERK1* site with an NGG PAM.
gOsCERK1-R4	aaacGTATACATACATCGGGACAc	
gOsJAR2-F3	GTGTGGATCCTCGCCGAGAACGG	Targeting the *OsJAR2* site with an NGG PAM.
gOsJAR2-R3	AAACCCGTTCTCGGCGAGGATCC	
gOsBRI1-F1	tgttgCCATTGCAGAATCTCACCC	Targeting the *OsBRI1* site with an NGG PAM.
gOsBRI1-R1	aaacGGGTGAGATTCTGCAATGGc	
gOsBZR1-F3	gtgtGCACCCGGACACGATACCG	Targeting the *OsBZR1-T1* site with an NGG PAM.
gOsBZR1-R3	aaacCGGTATCGTGTCCGGGTGC	
gOsFLS2-F3	GTGTGGATCCTCGCCGAGAACGG	Targeting the *OsFLS2* site with an NGG PAM.
gOsFLS2-R3	AAACCCGTTCTCGGCGAGGATCC	
gOsWRKY45-F12	tgttgGGAGCTACGACGCCGTCGC	Targeting the *OsWRKY45-T1* site with an NGG PAM.
gOsWRKY45-R12	aaacGCGACGGCGTCGTAGCTCCc	
gOsGS295-F2	gtgtgCTCGTGCCTGCCGGTGAGC	Targeting the *OsGS1-T1* site with an NGG PAM.

gOsGS295-R2	aaacGCTCACCGGCAGGCACGAGc	
gOsGS2-172-F1	tgttgGGATCACTGAATACTTGTG	Targeting the *OsGS2* site with an NAG PAM.
gOsGS2-172-R1	aaacCACAAGTATTCAGTGATCCc	
gOsBZR1-FAC	gtgtgCCTACAACCTCGTCAACCC	Targeting the *OsBZR1-T2* site with an NGG PAM.
gOsBZR1-RAC	aaacGGGTTGACGAGGTTGTAGGc	
gOsJAR1-FAC	tgttgTGCACACACAATGGGATGC	Targeting the *OsJAR1* site with an NGG PAM.
gOsJAR1-RAC	aaacGCATCCCATTGTGTGTGCAc	
gOsCOI2-F7	gtgtGTCGACCCTGCACCAGTGGC	Targeting the *COI2-T1* site with an NGC PAM.
gOsCOI2-R7	aaacGCCACTGGTGCAGGGTCGAC	
gOsSPL7-F1	tgttgGCACCTCCGACGCGTGGAC	Targeting the *SPL7* site with an NCC PAM.
gOsSPL7-R1	aaacGTCCACGCGTCGGAGGTGCc	
gOsWXb-F2	tgttgTACCTCAGCCACAACGCTG	Targeting the *OsWx* site in NTA PAM.
gOsWXb-R2	aaacCAGCGTTGTGGCTGAGGTAc	
gOsCOI2-F8	gtgtgGAGCGCGTCGACCCTGCAC	Targeting the *COI2-T2* site with an NAG PAM.
gOsCOI2-R8	aaacGTGCAGGGTCGACGCGCTCc	
gOsCOI2-F3a	TGTTGTCGACCCTGCACCAGTGG	Targeting the *COI2-T3* site with an NGG PAM.
gOsCOI2-R3a	AAACCCACTGGTGCAGGGTCGAC	

gOsALS1-G628-F1	tgttGCGCCCCCACTTGGGATCAT	Targeting the *OsALS1* site with an NGG PAM.
gOsALS1-G628-R1	aaacATGATCCCAAGTGGGGGCGC	
gOsAOS1-F1	tgttgGGCCGCGACGGCTTCTTCG	Targeting the *AOS1* site with an NGG PAM.
gOsAOS1-R1	aaacCGAAGAAGCCGTCGCGGCCc	
gOsGS-147-F1a	tgttgGCCACCAACAGGCCAGCCA	Targeting the *OsGS1-T2* site with an NGG PAM.
gOsGS-147-R1a	aaacTGGCTGGCCTGTTGGTGGCc	
gOsWRKY45-F13	tgttGATCACCGAAGAATCATGGA	Targeting the *OsWRKY45-T2* site with an NGG PAM.
gOsWRKY45-R13	aaacTCCATGATTCTTCGGTGATC	
gOsTUB-F2	GTGTGAAAGCATGAAGTGGATCCT	Targeting the *OsTubA2* site with an NGG PAM.
gOsTUB-R2	AAACAGGATCCACTTCATGCTTTC	
gOsAccase-F1	tgttgATAGCACTCAATGCGGTCT	Targeting the *OsACC1-T1* site with an NAA PAM.
gOsAccase-R1	aaacAGACCGCATTGAGTGCTATc	
gOsACCase-1999-F1	tgttgACCACACTTGTCCAGCACG	Targeting the*OsACC1-T2* site with an NGG PAM.
gOsACCase-1999-R1	aaacCGTGCTGGACAAGTGTGGTc	
HT-gOsCERK1-F4	ggagtgagtacggtgtgcGCACTAGATTCAGCAAGAGG	Detecting nucleotide changes of the *OsCERK1* site by Hi-TOM analysis.
HT-gOsCERK1-R4	gagttggatgctggatggCTGCAACCTGAAAGGTATAC	
HT-gOsJAR2-F3	ggagtgagtacggtgtgcTGACGCGCGACGCGGCCAAT	Detecting nucleotide changes of *OsJAR2* site by

HT-gOsJAR2-R3	gagttggatgctggatggAGGCGTCGCCGTCGGCGATG	Hi-TOM analysis.
HT-gOsBRI1-F1	ggagtgagtacggtgtgcTGGAGGCAAAATCTCTCCGG	Detecting nucleotide changes of the *OsBRI1* site by Hi-TOM analysis.
HT-gOsBRI1-R1	gagttggatgctggatggCAACCTTCCCATCCTTGAGC	
HT-gOsBZR1-F3	ggagtgagtacggtgtgcatcaggaagccggactggg	Detecting nucleotide changes of *OsBZR1-T1* site by Hi-TOM analysis.
HT-gOsBZR1-R3	gagttggatgctggatggTCGTGGCCATCTGGAAGCTG	
HT-gOsFLS2-F3	ggagtgagtacggtgtgcCGTCGCGGTGGACGGTCAGG	Detecting nucleotide changes of the *OsFLS2* site by Hi-TOM analysis.
HT-gOsFLS2-R3	gagttggatgctggatggACGCCCAGCATGCGCGCGGT	
HT-gWRKY45-F12	ggagtgagtacggtgtgcTCCCTTCGGGCCGGACCAGG	Detecting nucleotide changes of *OsWRKY45-T1* site by Hi-TOM analysis.
HT-gWRKY45-R12	gagttggatgctggatggCACGCGTGGAATCCATCTTC	
HT-gOsGS295-F2	ggagtgagtacggtgtgcTCAAGCTCAGGCACAAGGAG	Detecting nucleotide changes of the *OsGS1-T1* site by Hi-TOM analysis.
HT-gOsGS295-R2	gagttggatgctggatggAGGTGTTTTTCATGATCGGG	
HT-gOsGS1-147-F1a	ggagtgagtacggtgtgcTATCGCAACGTGTCACAGCC	Detecting nucleotide changes of *OsGS1-T2* site by Hi-TOM analysis.
HT-gOsGS1-147-R1a	gagttggatgctggatggGCCACAATCAAACAGACAAATG	
HT-gOsBZR1-FAC	ggagtgagtacggtgtgcGGTGGATCAGCTTCCAGATG	Detecting nucleotide changes of *OsBZR1-T1* site by Hi-TOM analysis.
HT-gOsBZR1-RAC	gagttggatgctggatggGAGCGTGAGCTCGAGCTCCT	
HT-gOsJAR1-FAC	ggagtgagtacggtgtgcGAGTACCTGCAGAACTTTGG	Detecting nucleotide changes of *OsJAR1* site by Hi-TOM analysis.
HT-gOsJAR1-RAC	gagttggatgctggatggCAAGCAATGGGTACAGTACC	

HT-gOsCOI2-F7	ggagtgagtacggtgtgcTGGTGATGGGGTTCGTGGAG	Detecting nucleotide changes of the *OsCOI2-T1~3* site by Hi-TOM analysis.
HT-gOsCOI2-R7	gagttggatgctggatggTTGAGCTTGAGCGACTCGAG	
HT-gOsSPL7-F1	ggagtgagtacggtgtgcGAGGGCAGCACGAGAATCAG	Detecting nucleotide changes of *OsSPL7* site by Hi-TOM analysis.
HT-gOsSPL7-R1	gagttggatgctggatggAGGTCGGACACTCAGACCAC	
HT-gOsWxb-F2	ggagtgagtacggtgtgcTGGTGATCTCTCCTCGGTAC	Detecting nucleotide changes of *OsWx* site by Hi-TOM analysis.
HT-gOsWxb-R2	gagttggatgctggatggCTCTCGTACCTGTCTGCAAC	
HT-OsALS-G628-F1	ggagtgagtacggtgtgcAGACTCCAGGGCCATACTTG	Detecting nucleotide changes of *OsALS1* site by Hi-TOM analysis.
HT-OsALS-G628-R1	gagttggatgctggatggCTTTATGGGTCATTCAGGTC	
HT-gOsAOS1-F3a	ggagtgagtacggtgtgcACCGGCAGGAGGTGGTGTCG	Detecting nucleotide changes of *OsAOS1* site by Hi-TOM analysis.
HT-gOsAOS1-R3a	gagttggatgctggatggTGTTGAGGCGCACCACCGTG	
HT-gOsGS1-147-F1a-2	ggagtgagtacggtgtgcTATCGCAACGTGTCACAGCC	Detecting nucleotide changes of *OsGS1-T2* site by Hi-TOM analysis.
HT-gOsGS1-147-R1a-2	gagttggatgctggatggGCCACAATCAAACAGACAAATG	
HT-gWRKY45-F13	ggagtgagtacggtgtgcGAACGACGAGGTTGTCTTCG	Detecting nucleotide changes of *OsWRKY45-T2* site by Hi-TOM analysis.
HT-gWRKY45-R13	gagttggatgctggatggCTAGCCAGGCTAATTAGGAG	
HT-gOsTUB-F2	ggagtgagtacggtgtgcTGACATCATGTGGCTAATGC	Detecting nucleotide changes of *OsTubA2* site by Hi-TOM analysis.
HT-gOsTUB-R2	gagttggatgctggatggTGATCTCCGCCACGGAGAGC	
HT-gOsACCase-F1	ggagtgagtacggtgtgcCTGGCTCGACTATTGTTGAG	Detecting nucleotide changes of *OsACC1-T1* site

HT-gOsACCase-R1	gagttggatgctggatggCAATTAACCCTTGCGGTTCC	by Hi-TOM analysis.
HT-gOsACC1999-F1	ggagtgagtacggtgtgcGGCTAAGACAGTGGTTACTG	Detecting nucleotide changes of *OsACC1-T2* site by Hi-TOM analysis.
HT-gOsACC1999-R1	gagttggatgctggatggGTTGAAGTCCAGCAATGCCT	
U6HT-F1	ggagtgagtacggtgtgcAGCGTTGAGGGGAGACAGG	Detecting self-editing of *OsSPL7* and *OsWx* site by Hi-TOM analysis.
U6HT-R1	gagttggatgctggatggCAGGGACCTGGTTGGAAATCTC	
U6HT-F2	ggagtgagtacggtgtgcGAGAGGCGGGAGGAACAGTT	Detecting self-editing of *OsCOI2-T2* and *OsCOI2-T1* site by Hi-TOM analysis.
U6HT-R2	gagttggatgctggatggATTGCCCTTCGAAGGGACAA	
